# Esterification of 3-quinuclidinol, a marker for the incapacitant BZ, for analysis by EI-GC-MS in OPCW test matrices

**DOI:** 10.1038/s41598-025-22716-6

**Published:** 2025-11-06

**Authors:** David S. Cho, David Baliu-Rodriguez, Carlos A. Valdez

**Affiliations:** 1 Physical and Life Sciences Directorate, Livermore, CA 94550 USA; 2 Forensic Science Center, Livermore, CA 94550 USA; 3 Global Security Directorate, Livermore, CA 94550 USA; 4 Biosciences and Biotechnology Division, L-090, 7000 East Avenue, Livermore, CA 94550 USA

**Keywords:** 3-quinuclidinol, 3-quinuclidinyl benzilate, Incapacitant, Acylation, Soil, Biochemistry, Chemistry

## Abstract

The analysis by EI-GC-MS of 3-quinuclidinol (3Q), a marker for the riot control and incapacitating agent quinuclinidyl benzilate (BZ), using several, new acylation strategies is described. After evaluating and optimizing conditions for the acylations, these were tested on their ability to successfully derivatize 3Q in three different matrices featured in the 44th Organisation for the Prohibition of Chemical Weapons (OPCW) proficiency test (PT). As 3Q is a highly polar compound, the work here describes acylation strategies that generate 3Q analogs with superior gas chromatographic profiles relative to the underivatized material. The acyl groups studied in this work included acetyl, benzoyl, pentafluorobenzoyl and the bis(3,5-trifluoromethyl)benzoyl. The acylated 3Q products provide sharp, detectable peaks with significantly different retention times from that of the unmodified 3Q. Thus, the retention times for acetyl-, benzoyl-, pentafluorobenzoyl- and 3,5-bis(trifluoromethyl)benzoyl-3Q were determined to be 15.9, 24.9, 22.8 and 22.1 min respectively, in stark contrast to the unmodified 3Q which provides a sharp peak with a retention time centered at ~ 13.5 min only at high concentrations (> 10 µg/mL or µg/g) while a broad peak (RT ~ 15–15.5 min) at low concentrations (< 5 µg/mL or µg/g). The developed protocol was used to derivatize 3Q in three separate matrices. The first two matrices were liquid samples featured during the 44th OPCW PT at two separate concentrations in each (5 and 50 µg/mL). The last matrix was a soil sample featured in the same 44th OPCW PT wherein the 3Q had been spiked at a 12 µg/g concentration. The approach involves the extraction of 3Q from all matrices followed by its acylation resulting in a second reportable analytical method for an OPCW PT. The presented derivatization strategies should find wide applicability particularly in laboratories involved with the analysis of this chemical weapon agent degradation product and those participating in the yearly OPCW PTs.

**Supplementary Information:**

The online version contains supplementary material available at 10.1038/s41598-025-22716-6.

## Introduction

The analysis and unambiguous identification of degradation products related to chemical warfare agents (CWAs) in the field is of key importance in providing clues to the past presence of these toxic chemicals in the environment^1^. Detection of these species either in their native or derivatized form provide crucial information to forensic teams conducting inspection on behalf of the chemical weapons convention (CWC). Initial assessment on the field is often carried out using portable instrumentation and to this end, gas chromatography-mass spectrometry (GC-MS) has become one of the most useful tools in this endeavor^2–5^. Most CWAs employed in theater do not persist for long periods of time because of their exposure to various environmental factors that accelerate their degradation (e.g., heat, moisture, plant and soil matter)^6,7^. For this reason, inspection teams working on behalf of the Organisation for the Prohibition of Chemical Weapons (OPCW)^8^ rely heavily on the detection of products arising from their degradation and, as such, have employed clever derivatization techniques to convert these polar species into derivatives that can be detected by GC-MS^9–11^. Alternatively, degradation can be brought upon deliberately on the CWA by various pathways including decontamination with high pH aqueous solutions as well as oxidative chemistries that accelerate their breakdown into other species despite the resistance to degradation by the original CWA^12,13^.

**Fig. 1:**
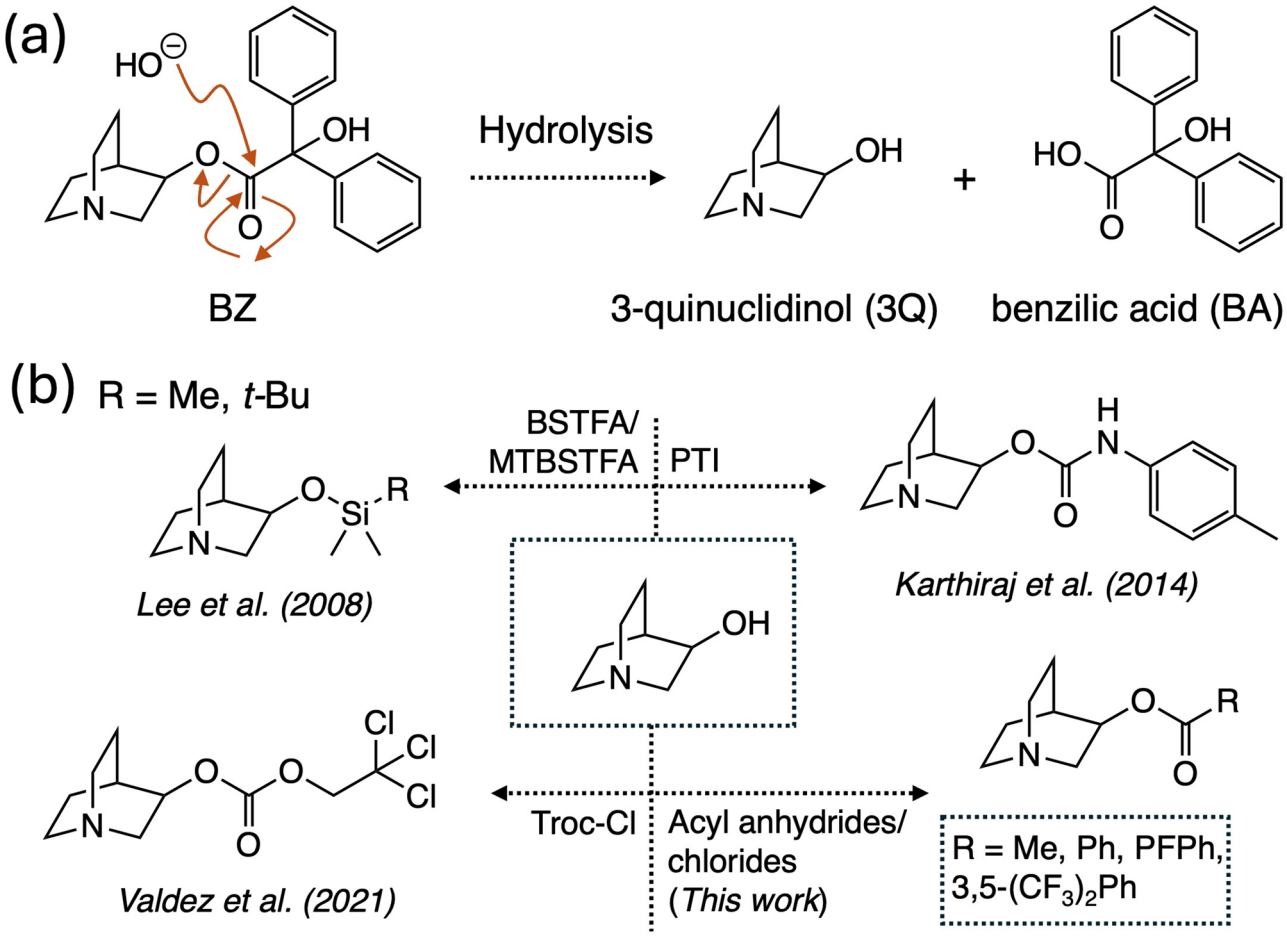
(**a**) Hydrolysis of BZ to yield 3Q and BA and (**b**) derivatization methods commonly devised for the modification of 3Q for GC-MS analysis such as carbamate formation with PTI (*p*-tolylisocyanate), silylation using BSTFA and MTBSTFA, carbonate formation using Troc-Cl. The use of acylating agents (anhydrides and chlorides) to give 3-*O*-acyl derivatives of 3Q is described in this work.

3-Quinuclidinyl benzilate (BZ) is a chemical belonging to the class of incapacitating agents under the CWA umbrella and classified as a Schedule 2 substance by OPCW^14–16^ (Fig. 1a). As an incapacitating agent, BZ acts on the muscarinic receptors and produces biological effects like hallucinations, irrational behavior and disorientation^17^, and can be lethal at large doses. One of the main structural features in BZ is a unique, bicyclic and abiotic 3-quinuclidinol (3Q) nucleus connected via an ester linkage to a benzilic acid (BA) unit (Fig. 1a). It is this sterically hindered ester linkage that gives rise to BZ’s long persistence in the environment but still yielding 3Q and BA as two products arising from a hydrolytic process. Due to the abiotic nature of 3Q, this material serves as an indicative marker for the past or current presence of BZ in environmental matrices such as ponds, rivers or soils. Due to its direct link to a Schedule 2 material, it is not surprising that 3Q has become a common analyte in matrices featured in proficiency tests (PTs) administered yearly by OPCW^18,19^.

To this end, several analytical methods can be used for its detection such as liquid chromatography-mass spectrometry (LC-MS)^20,21^ and GC-MS^9^. Due to its amino alcohol-based structure, 3Q, although detectable when present in large concentrations by GC-MS, features a broad peak that only serves to complicate its detection (RT ~ 15–15.5 min). This property creates challenges when the analysis of 3Q is taking place in a matrix where it is present at low concentrations (~ 10 µg/g) and/or co-eluting with more abundant interferences. Therefore, several strategies to not only improve the chromatographic profile of 3Q (i.e., peak sharpness) but also to shift its retention time to longer values to avoid co-elution with interferences have been foci of extensive research among analytical chemistry groups. One of the first ones was to employ silylation to form trimethylsilyl and *tert*-butyldimethylsilyl versions of 3Q (i.e., 3Q-*O*-TMS and 3Q-*O*-TBDMS) in situ while collecting the 3Q, along with other amino alcohols, via hollow fiber-liquid phase microextraction^22^ (Fig. 1b). Another strategy developed by Karthiraj et al. involves carbamate formation by reacting 3Q with *p*-tolylisocyanate^23^. This technique produces a highly stable 3-*O*-carbamate product that greatly increases the retention time of 3Q away from early eluting interferences and additionally provides an abundant molecular ion peak for further identification (*m/z* = 260, 51%) (Fig. 1b). A third strategy for derivatization for 3Q, introduced by our group, is trichloroethoxycarbonylation using 2,2,2-trichloroethoxycarbonyl chloride (Troc-Cl) to generate a carbonate derivative with longer retention time (RT = 23.8 min.) and improved chromatography^24^ (Fig. 1b).

In this work, we introduce acylation as a reliable derivatization alternative for the analysis of 3Q for OPCW PT purposes as well as its analysis in complex environmental matrices. The strategy involves the acylation of the 3Q using optimized conditions to introduce ester moieties that confer these new 3Q derivatives with improved GC-MS characteristics relative to the native 3Q such as improved chromatography in the form of sharper peaks as well as different retention times. We have explored the use of four acylating agents: acetic anhydride, benzoyl chloride, pentafluorobenzoyl chloride and *bis*(3,5-trifluoromethyl)benzoyl chloride to yield four different 3Q derivatives with various molecular weights and unique GC-MS profiles. Furthermore, we have tested the approach in the successful analysis of 3Q in three different matrices, one soil (S1) and two liquid, aqueous ones (L1 and L2), featured during the 44th OPCW PT spiked at 12 µg/g, and 50 µg/mL and 5 µg/mL respectively.

## Materials and methods

*Chemicals.* All chemicals were purchased from commercial suppliers and used as received. (±) −3-Quinuclidinol was purchased from BeanTown chemicals (Hudson, NH.). Anhydrous dichloromethane (DCM), acetic anhydride, benzoyl chloride, pentafluorobenzoyl chloride and 3,5-*bis*(trifluoromethyl)benzoyl chloride were purchased from Sigma-Aldrich (St. Louis, MO.). Sodium carbonate, sodium hydrogen carbonate (NaHCO_3_) and anhydrous sodium sulfate were purchased from Acros Organics (Westchester, PA.). Autosampler vials and glass inserts were purchased from Agilent Technologies (Santa Clara, CA.). The standards 3-*O*-acetyl-3-quinuclidinol (3Q-Ac), 3-*O*-benzoyl-3-quinuclidinol (3Q-Bz), 3-*O*-pentafluorobenzoyl-3-quinuclidinol (3Q-PFBz) and 3-*O*-[*bis*(3,5-(trifluoromethyl))]benzoyl-3-quinuclidinol (3Q-BTFMBz) were synthesized in house following standard acylation protocols^25^. For the synthesis of the standards, thin layer chromatography (TLC) was used to monitor the course of the acylations using Agela Technologies glass back MF_254_ TLC plates (pH ~ 5) and detection of products was accomplished with UV light (λ = 254 nm) in conjunction with development of color with ceric ammonium molybdate (CAM) and iodine vapor^26,27^. Wheaton scintillation vials (20-mL capacity) and glass vials (4-mL capacity) were purchased from VWR (Radnor, PA). All acylated standards were purified by flash column chromatography using a Biotage Isolera purification system using Biotage Sfär silica high-capacity duo cartridges (10 G) using a gradient of DCM → 10% MeOH/DCM over six column volumes.

### Synthesis of 3-O-acyl/benzoyl-3Q standards

**Fig. 2:**
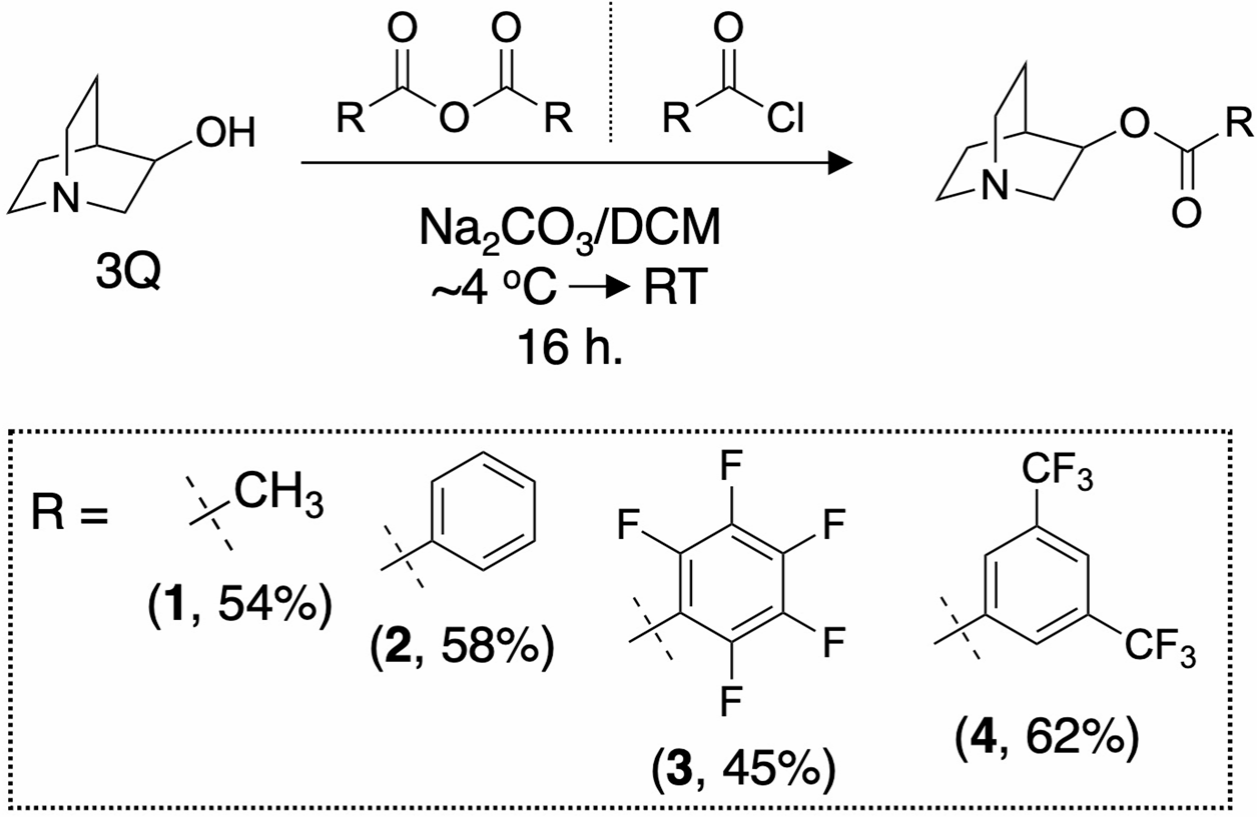
Chemical synthesis of all 3Q standards used in this work. For 3Q-Ac (**1**), acetic anhydride was used while for the remaining acyl derivatives **2**–**4**, the respective acyl chlorides were used.

General Procedure. 3-Quinuclidinol (300 mg, 2.36 mmol) was dissolved in anhydrous DCM (20 mL) in a 50-mL round bottom flask equipped with a stir bar. To the colorless solution, sodium carbonate (312 mg, 2.96 mmol, 1.25 equiv. to 3Q) was added in one portion and then cooled using an ice bath (~ 4 °C). The cooled solution was treated dropwise with the acyl chloride/anhydride reagent (2.60 mmol, 1.1 equiv. to 3Q). After the addition of the acylating reagent, the resulting suspension was allowed to warm to ambient temperature and stirring was continued overnight. The following day, the mixture was transferred to a 250-mL separatory funnel and partitioned (DCM//H_2_O). The organic phase was washed with NaCl/H_2_O (2 × 10 mL), dried over anhydrous sodium sulfate, evaporated *in vacuo* to yield a light amber oil that was purified by flash column silica gel chromatography using a Biotage Isolera unit (DCM → 10% MeOH/DCM) to furnish the acylated 3Q products as white solids or colorless oils. The synthetic procedures are shown on Fig. 2 below.

3Q-Ac (**1**). Synthesized according to the general procedure using acetic anhydride (246 µL) and obtaining **1** as a white solid (215 mg, 54%) and found to be identical to the published one^28^. HRMS calculated for C_9_H_16_NO_2_ [M + H]^+^: 170.1176, found: 170.1174.

3Q-Bz (**2**). Synthesized according to the general procedure using benzoyl chloride (440 µL), obtaining **2** as a colorless oil (316 mg, 58%). and found to be identical to the published one^29^. HRMS calculated for C_14_H_18_NO_2_ [M + H]^+^: 232.1332, found: 232.1331.

3Q-PFBz (**3**). Synthesized according to the general procedure using pentafluorobenzoyl chloride (374 µL), obtaining **3** as a thick, colorless oil (341 mg, 45%). ^1^H NMR (CDCl_3_, 600 MHz) δ 5.34–5.31 (m, 1H), 3.64 (ddd, *J* = 14.7, 8.7, 2.0 Hz, 1H), 3.31–3.16 (m, 5 H), 2.47–2.45 (m, 1H), 2.19–2.12 (m, 1H), 2.05–1.99 (m, 1H), 1.91–1.84 (m, 1H), 1.80–1.75 (m, 1H); ^13^C NMR (CDCl_3_, 150 MHz) δ 164.2 (C = O), 158.3, 146.6-146.4.6.4 (m), 144.9-144.3.9.3 (m), 143.0-142.6.0.6 (m), 141.6-141.3.6.3 (m), 139.9-139.6.9.6 (m), 138.7-138.5.7.5 (m), 138.3–138.0 (m), 137.1-136.8.1.8 (m), 136.6-136.4.6.4 (m), 115.6-115.2.6.2 (m), 107.4-107.1.4.1 (m), 70.8 (C-H), 53.2, 46.1, 45.2, 24.5 (C-H), 21.7, 17.8; R_f_ (10% MeOH/DCM) = 0.55; HRMS calculated for C_14_H_13_F_5_NO_2_ [M + H]^+^: 322.0861, found: 322.0863.

3Q-TFMBz (**4**). Synthesized according to the general procedure using 3,5-bis(trifluoromethyl)benzoyl chloride (472 µL) obtaining **4** as a colorless oil (537 mg, 62%). ^1^H NMR (CDCl_3_, 600 MHz) δ 8.46 (s, 1H), 8.07 (s, 1H), 5.14–5.11 (m, 1H), 3.41 (ddd, *J* = 14.7, 8.7, 2.1, 1H), 3.04–2.99 (m, 1H), 2.99–2.93 (m, 1H), 2.92–2.79 (m, 3 H), 2.22–2.19 (m, 1H), 1.98–1.92 (m, 1H), 1.82–1.77 (m, 1H), 1.70–1.64 (m, 1H), 1.57–1.51 (m, 1H); ^13^C NMR (CDCl_3_, 150 MHz) δ 163.6 (C = O), 132.5, 132.2 (q, *J*_C−F_ = 33.8 Hz), 129.6 (C-H), 129.6 (C-H), 126.4-126.3.4.3 (m, C-H), 122.8 (q, *J*_C−F_ = 273.3 Hz), 73.3 (C-H), 55.2, 47.3, 46.3, 25.3 (C-H), 24.3, 19.5; R_f_ (10% MeOH/DCM) = 0.48; HRMS calculated for C_16_H_16_F_6_NO_2_ [M + H]^+^: 368.1080, found: 368.1091.

### EI-GC-MS analysis method

A 6890 Agilent GC with a 5975 MS detector equipped with a split/splitless injector was used for all analyses as previously described^30,31^. The GC column used for the analyses was an Agilent HP-5ms UI capillary column (30 m × 0.25 mm id × 0.25 μm film thickness). Ultra-high purity helium, at a 0.8 mL/min flow, served as the carrier gas. The inlet was operated in pulsed splitless mode (25 psi for 1 min, followed by a 50 mL/min purge flow). The injector temperature was set at 250 °C and the injection volume used was 1 µL. The oven temperature program was as follows: 40 °C, held for 3 min, increased at 8 °C/min to 300 °C and held at this temperature for 3 min. The MS ion source and quadrupole temperatures were 230 °C and 150 °C, respectively. Electron impact (EI) was used with an ionization energy of 70 eV. The MS was operated to scan from *m/z* = 29 to 600 in 0.4 s with a solvent delay of 3.5 min.

### Nuclear magnetic resonance

^1^H NMR (600 MHz), ^13^C NMR (150 MHz) and^19^F NMR (565 MHz) were all recorded in CDCl_3_. Spectra were obtained using a Bruker Avance III 600 MHz instrument equipped with a Bruker QNP 5 mm cryoprobe (Bruker Biospin, Billerica, MA) at 25.0 ± 0.1 °C. ^1^H NMR data is reported as follows: chemical shift (δ) (parts per million, ppm); multiplicity: ddd (doublet of doublets of doublets) and m (multiplet); coupling constants (*J*) are given in Hertz (Hz). ^1^H NMR chemical shifts are calibrated with respect to residual CHCl_3_ centered at 7.26 ppm. ^13^C NMR chemical shifts are calibrated to the center peak for CDCl_3_ at 77.0 ppm. ^13^C NMR-DEPT-135 data is presented so that positive peaks depict C-H and CH_3_ carbons atoms while negative peaks depict CH_2_ carbon atoms in the molecule (See Supporting Information). Quaternary carbons (e.g., C = O for the acyl group) are not observed in the^13^C NMR-DEPT-135 experiment.

## Results and discussion

Incapacitating agents form part of a unique class of toxic compounds within the field of CWAs^10,11^. While some like the organophosphorus-based nerve agents (OPNAs) are highly toxic and originally designed to rapidly eliminate a threat, others like the incapacitating agents are used to non-lethally subdue a threat. With regards to the incapacitating agents, some members in this class that are effective but can also lethal when not used correctly are the fentanyls^32^ as highlighted by their use in the Dubrovka theater siege by the Russia military to effectively subdue Chechen terrorists but in the process causing the death of 132 civilians^33,34^. Another member in this family is 3-quinuclidinyl benzilate (QNB) or BZ by its NATO acronym^35^. Much like the fentanyls and the other nervous system-targeting incapacitants, BZ acts upon the nervous system by blocking nerve impulses from reaching various vital organs^36,37^. It is this disruption of biological signaling events that results in muscle relaxation leading to respiratory arrest if not treated immediately with an opioid antagonist such as Naloxone in the case of the fentanyls^38,39^or tacrine and its analogs in the case of BZ^40^. BZ has been subject of study for detection and identification by various analytical methods that mainly included LC-based hyphenated techniques^41,42^. Within the context of GC-MS, although BZ can be detected in intact form^43^, a great deal of effort has gone into the analysis of its two hydrolysis products: 3Q and BA (Fig. 1a)^10^.

Naturally, due to their link to a schedule 2 substance, 3Q and BA are commonly spiked materials featured during PTs administered yearly by OPCW^16^. Both are polar products themselves and as a such provide their own difficulties when analyzed by GC-MS means. In the case of BA, derivatization techniques have been applied to its analysis by GCMS^10,44^. Similarly, 3Q has been the subject of many clever derivatization approaches to furnish analogs with reduced polarity for successful GC-MS detection. During OPCW PTs, participating laboratories need to correctly identify specific analytes that have been spiked in various matrices at concentrations that range anywhere from 1 to 50 ppm. Using a combination of efficient extractions followed by a suitable derivatization, some analytes bearing polar groups can be detected in derivatized form that enhances their detection by GC-MS. For this work, matrices featured during the 44th OPCW PT were used to illustrate the utility of the derivatization reactions. The scenario presented in this particular PT was that the matrices consisted of aqueous decontamination solutions and soil samples collected near an area suspected of serving as a chemical storage facility. Another interesting facet encountered during this PT was that for the first time the same reportable spiking chemical, 3Q, was spiked at 50, 12, and 5 ppm concentrations in the three distinct matrices.

**Fig. 3:**
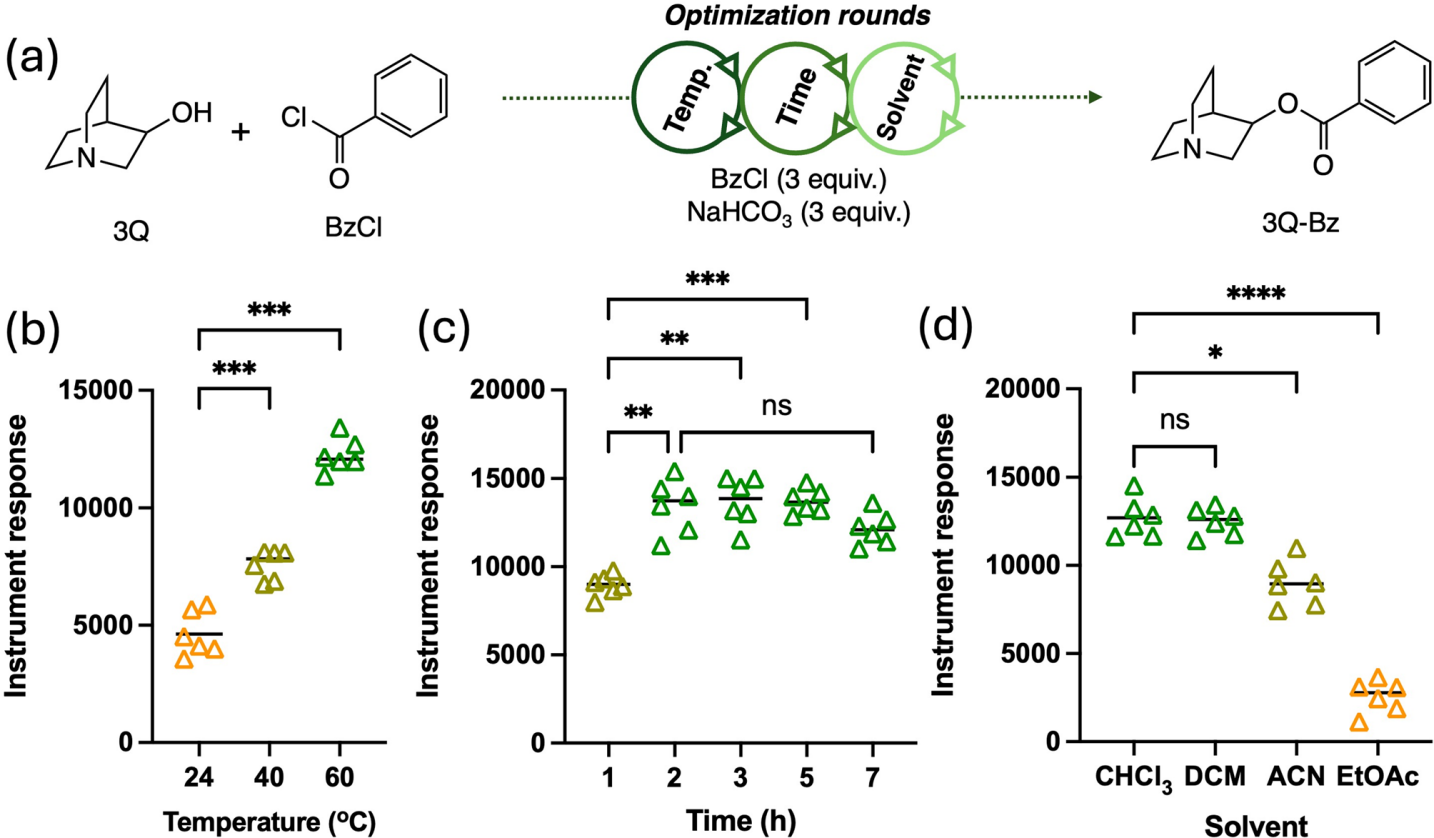
(**a**) Optimization protocols on reaction parameters carried out on the benzoylation of 3Q as a model reaction that included (**b**) temperature, (**c**) time and (**d**) solvent medium. Average (*n* = 6) peak areas (+/- the standard deviation), statistical analysis using one-way ANOVA followed by Tukey’s multiple comparison’s test analysis revealed significance at a level of ***p = 0.0004 (temperature) and ***p = 0.0005 (time); **p = 0.0036 (time) and ****p < 0.0001 and *p = 0.0145 (solvent).

After the success of our previous work involving pinacolyl alcohol (PA)^25^, our group decided to explore acylation as a viable strategy for the chemical modification of 3Q and subsequent analysis by GC-MS. A characteristic that makes 3Q and other CWA-related aminoalcohols such as the *N*,* N*-dialkylethanolamines challenging analytes to derivatize efficiently is the presence of a basic nitrogen in their structures that can compete with derivatization processes such as alkylation with the oxygen center^45,46^. To this end, it was decided that the focus of this work will be centered on performing derivatizations on 3Q using four acylating agents, namely: acetic anhydride, benzoyl chloride, pentafluorobenzoyl chloride and 3,5-*bis*-trifluoromethylbenzoyl chloride. It was established that the use of the derivatization agent would be in large excess when applying it to real samples for analysis, so efforts were focused on determining the effects of temperature, time and solvent medium on the reaction’s efficiency (Fig. 3). The model reaction system used for the optimization studies was the one involving the benzoylation of 3Q in the presence of NaHCO_3_. The purpose of using NaHCO_3_ as a base, over other organic ones like triethylamine, is to limit the introduction of additional signals during the GC-MS analysis due to the insolubility of the salt as well as its low basicity which minimizes any minor hydrolysis of other analytes of interest in the matrix.

The effect of temperature on the reaction was found to be that of an accelerating nature reaching out maximum yields within an hour when the reaction was heated to 60 °C (Fig. 3a). However, conducting the derivatization at ambient temperature with no need of heating yields the same result within 2 h which has the added benefit of not potentially destroying any other analytes of interest in a mixture. Regarding the time of the reaction, seems like at room temperature, most of the 3Q has already being converted to its acylated form within the first 2 h with prolonged reaction time not having a significant impact on product yield (Fig. 3b). The last element of optimization was the nature of the solvent for conducting the derivatization. We evaluated four solvents based on their common use for GC-MS analyses and also for their ability to promote the acylation reaction as a result of their innate polarities that can be beneficial to the stabilization of intermediates generated during the reaction. The four solvents evaluated were dichloromethane, chloroform, acetonitrile and ethyl acetate (Fig. 3c). It was found that although acetonitrile and ethyl acetate do seem to benefit the formation of the product, the same is true for dichloromethane and chloroform upon prolonged reaction time. Due to the generality of these chlorinated solvents in GC-MS analyses and also for their low boiling point particularly in the case of DCM, we decided to choose this solvent for our subsequent experiments nevertheless remarking that acetonitrile and ethyl acetate remain suitable solvents for the derivatization.

As it is the case in every PT, confirmation of the spiked, reportable compounds must be done in accordance to OPCW reporting rules involving mostly the use of standards that have been synthesized or purchased. Aside from 3Q-PFBz and 3Q-TFMBz, which were chosen to illustrate the use of acylation strategies in the analysis of 3Q in OPCW PT-related matrices, 3Q-Ac and 3Q-Bz were also synthesized in house and characterized by ^1^H, ^13^C, ^13^C-DEPT135 NMR and EI-GC-MS. The EI-GC-MS chromatographic and mass spectral data are summarized in Table 1.

**Table 1:** EI-GC-MS spectral data for all four 3Q acyl standards prepared in this work.

3Q-Derivative Name (abbreviated code) [Mol. Formula]	Retention time (min)^1^	Chemical structure	EI-GC-MS fragments (% of base peak)^2,3,4^	RI^5^
3Q-acetyl (3Q-Ac) [C_9_H_15_NO_2_]	15.83		**126**^†^, 169* (37), 42 (27), 43 (25), 98 (25), 127 (22), 110 (16), 109 (13), 55 (12), 70 (11).	1314
3Q-benzoyl (3Q-Bz) [C_14_H_17_NO_2_]	24.91		**126**^†^, 105 (41), 77 (39), 231* (33), 203 (21), 109 (17), 98 (16), 42 (14), 51 (12), 55 (9).	1938
3Q-pentafluorobenzoyl (3Q-PFBz) [C_14_H_12_F_5_NO_2_]	22.82		**126**^†^, 195 (45), 321* (30), 42 (24), 167 (23), 98 (17), 110 (15), 117 (14), 55 (11), 293 (10).	1769
3Q-[3,5-bis(trifluoromethyl)] benzoyl (3Q-BTFMBz) [C_16_H_15_F_6_NO_2_]	22.17		**126**^†^, 241 (27), 213 (27), 367* (21), 42 (18), 348 (17), 98 (13), 339 (12), 110 (11), 109 (9).	1725

^1^HP-5ms UI capillary column (30 m × 0.25 mm id × 0.25 μm film thickness), total run time = 40 min.

^2^The ten most intense peaks are provided along with their intensity relative to the base peak (m/z = 126).

^3^Molecular ion peaks denoted with an *.

^4^Base peaks denoted with a † and bold-faced.

^5^RI values calculated using *n*-alkanes standard (C8-C36), Restek 573,771 (dilution to 8 ppm in DCM).

## GC-MS analysis of 3Q in soil matrix featured in OPCW PT (44th)

**Fig. 4:**
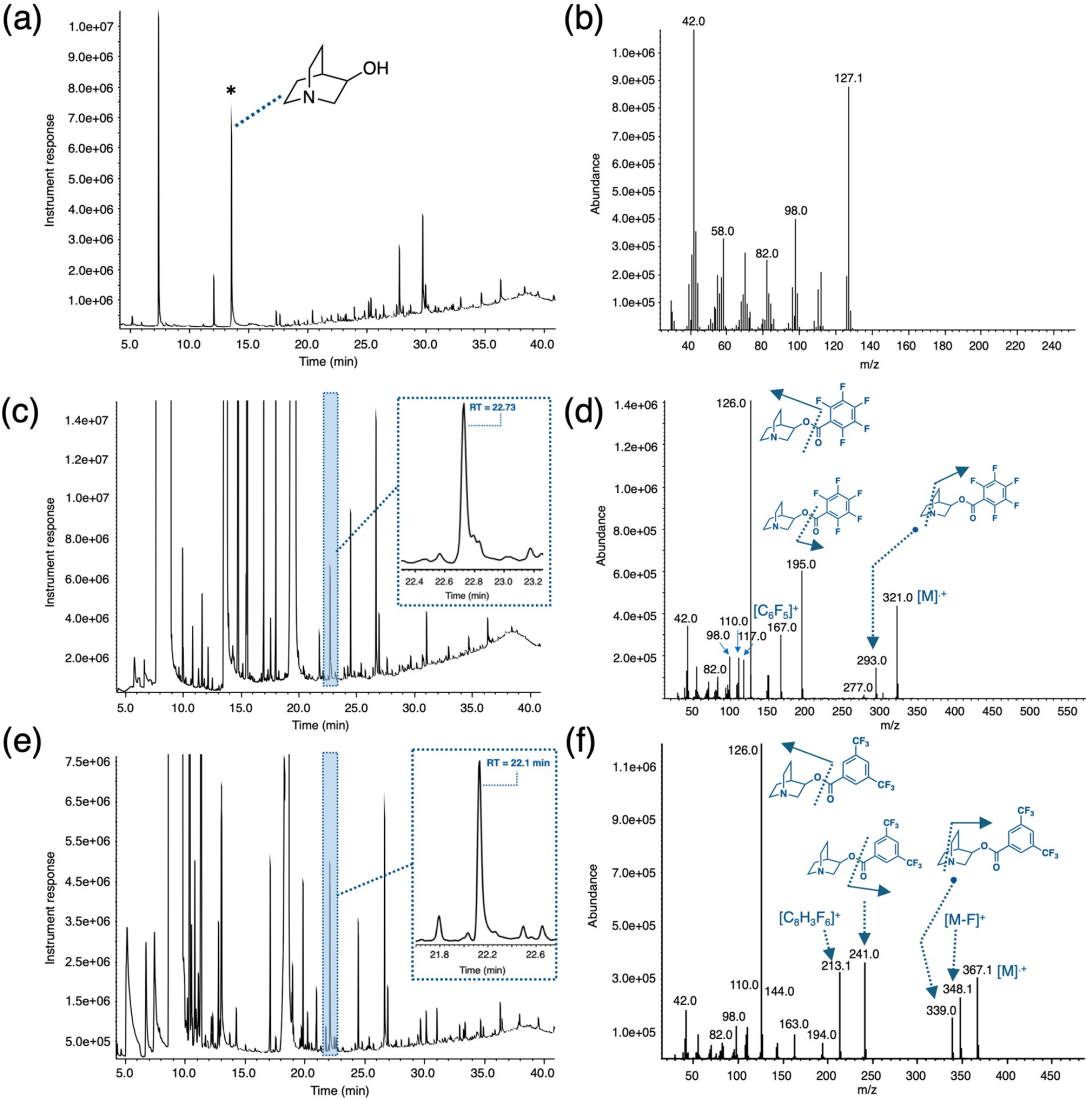
(**a**) TIC for analysis of extracted soil matrix S1 where 3Q is spiked at 12 ppm concentration along with its (**b**) mass spectrum showing its strong molecular peak (m/z = 127),(**c**) TIC obtained after the reaction of the soil extract S1 with pentafluorobenzoyl chloride showing the formation of the 3Q-PFBz with a retention time of 22.8 min (inset) and its (**d**) associated mass spectrum that still displays the molecular ion peak at m/z = 321; (**e**) TIC obtained after the reaction of the soil extract S1 with 3,5-bis(trifluoromethyl)benzoyl chloride showing the formation of the 3Q-BTFMBz with a retention time of 22.1 min (inset) and its (**f**) associated mass spectrum that still displays the molecular ion peak at m/z = 367.

The soil matrix (S1) featured in the PT was granular in consistency and initial analysis by EI-GC-MS showed that it consisted of some organics (terpenes) and 3Q as one of the reportable chemicals spiked at a concentration of 12 µg/g. At this concentration and given the relatively low concentration of the organics, the signal for 3Q is clear (RT = 13.6 min) and sharp after its extraction from the soil along with other constituents using DCM (Fig. 4a). The matrix was taken up in DCM and treated with excess pentafluorobenzoyl chloride (PFBzCl) for 2 h at ambient temperature to yield 3Q-OPFBz in the matrix. The 3Q-PFBz features a longer retention time (RT = 22.7 min) relative to 3Q, and it can be detected as a sharp peak and away from other interferences in the matrix including the PFBzCl that was used in excess (Fig. 4c). Additionally, analysis of the 3Q-PFBz reveals a distinct mass spectrum where a clear molecular ion peak can be observed (Fig. 4d). Regarding the mass spectrum of 3Q-PFBz, one of the main features is the presence of a strong molecular ion peak (m/z = 321) and the presence of a distinctive base peak m/z = 126 arising from the fragmentation of 3Q-PFBz at the ester bond (Fig. 4d; Table 1). The base peak at m/z = 126 functions as a diagnostic tool when selectively extracting ions during spectrum analysis. Other important ions that can be identified are m/z = 293 which arises from the loss of ethylene from the bicyclic unit of 3Q-PFBz as well as m/z = 195 which arises from the fragmentation of 3Q-PFBz at the ester bond yielding a [C_7_F_5_O]^+^ fragment. Other diagnostic fragments include m/z = 167, arising from the pentafluorotropyllium [C_6_F_5_]^+^ cation and m/z = 110 corresponding to the quinuclidine nucleus [C_6_H_12_N].^+^.

## GC-MS analysis of 3Q in liquid matrix 1 featured in OPCW PT (44th)

**Fig. 5:**
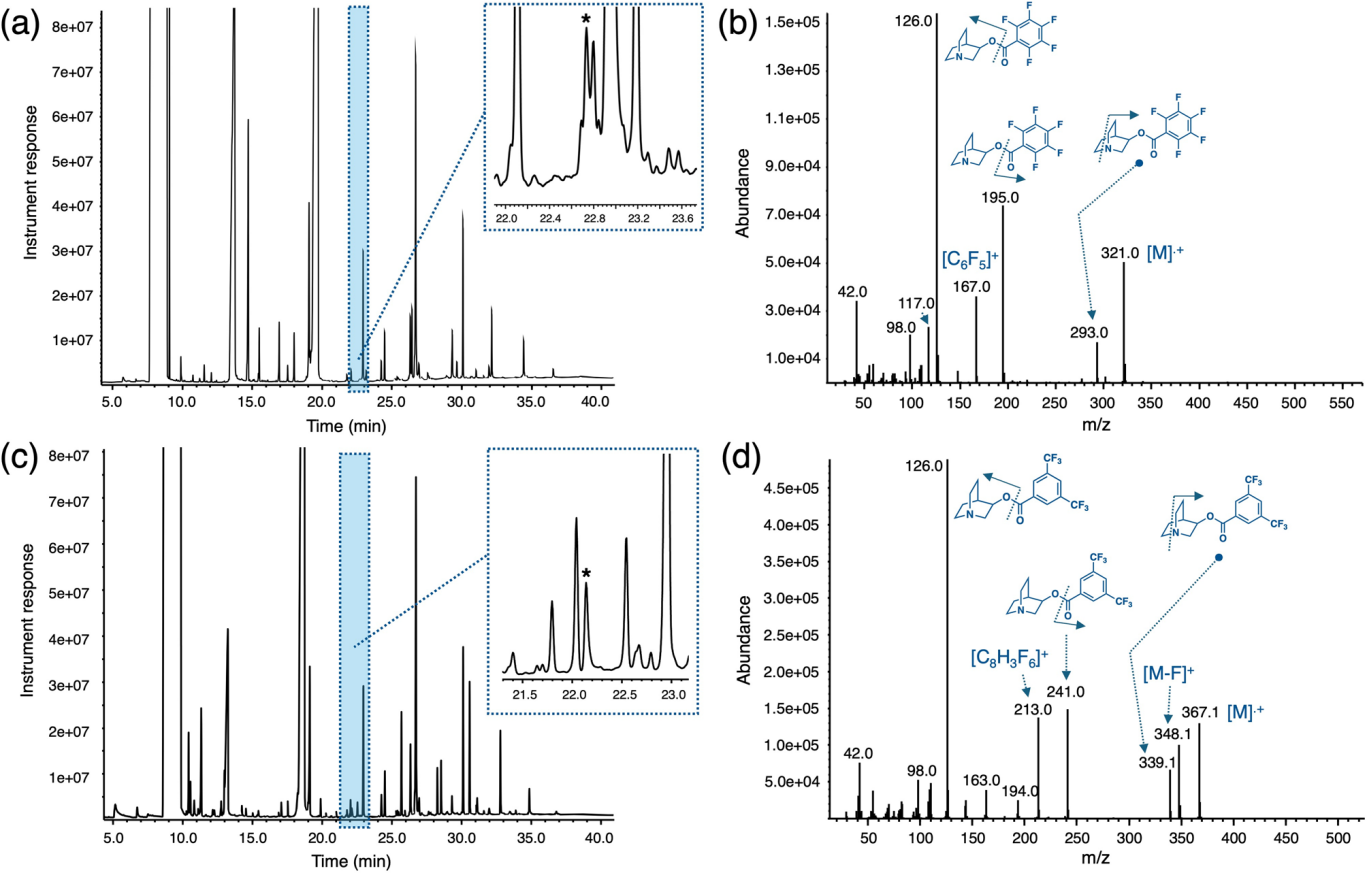
(**a**) Pentafluorobenzoylation of extract from L1 aqueous matrix (3Q spiked at a ~ 5 ppm concentration) to yield derivative 3Q-PFBz featuring a retention time of 22.78 min (denoted with an asterisk in inset); (**b**) mass spectrum of 3Q-PFBz from the derivatized mixture showing proposed fragmentation patterns for specific masses; (**c**) 3,5-bis(trifluoromethyl)benzoylation of extract from L1 aqueous matrix to yield derivative 3Q-BTFMBz featuring a retention time of 22.14 min (denoted with an asterisk in inset); (**d**) mass spectrum of 3Q-BTFMBz from the derivatized mixture showing proposed fragmentation patterns for specific masses.

Liquid matrix 1 (L1) was an aqueous based solution of various alcohols and organics that contains the 3Q spiked at a ~ 5 µg/mL concentration. The nature of the alcohols in the mixture was determined to be low-medium molecular weight species ranging from C4 to C7 and in some cases as tentatively assigned using the NIST library, possessing degrees of unsaturation in the form of double bonds or ring systems. The remaining organics were found to be, again tentatively assigned by library matching, low molecular weight carboxylic acid methyl esters and phenol-based compounds. It is in this mixture that 3Q was spiked at the lowest concentration for the PT. After performing an extraction of the aqueous mixture with DCM, the extract was evaporated with a nitrogen stream and then derivatized using both fluorinated benzoyl reagents. The first derivatization involved reacting the mixture with pentafluorobenzoyl chloride to give 3Q-PFBz (RT ~ 22.78 min) that was detected without using the single ion extraction mode (Fig. 5a). It can be observed from Fig. 5a, that 3Q-PFBz can still be observed among some of the other interferences present in the L1 matrix and that were extracted along with 3Q, despite the low concentration of the amino alcohol in it. Figure 5b shows the mass spectrum of the 3Q-PFBz that can be obtained successfully by performing a background subtraction in the mass spectrum. The second derivatization involved reacting the mixture with 3,5-*bis*(trifluoromethyl)benzoyl chloride to give 3Q-BTFMBz (RT ~ 22.14 min) (Fig. 5c). Again, 3Q-BTFMBz can easily be detected without the need of the single ion extraction mode and its mass spectrum can be readily identified by performing background subtraction methods away from the interferences that are at similar or higher concentrations (Fig. 5d). One noteworthy observation is the slight deviation in retention time of both derivatives when produced in the L1 matrix. This could be attributed to matrix effects that can cause shifts in the retention times of analytes, particularly when these are present at low concentrations.

## GC-MS analysis of 3Q in liquid matrix 2 featured in OPCW PT (44th)

**Fig. 6:**
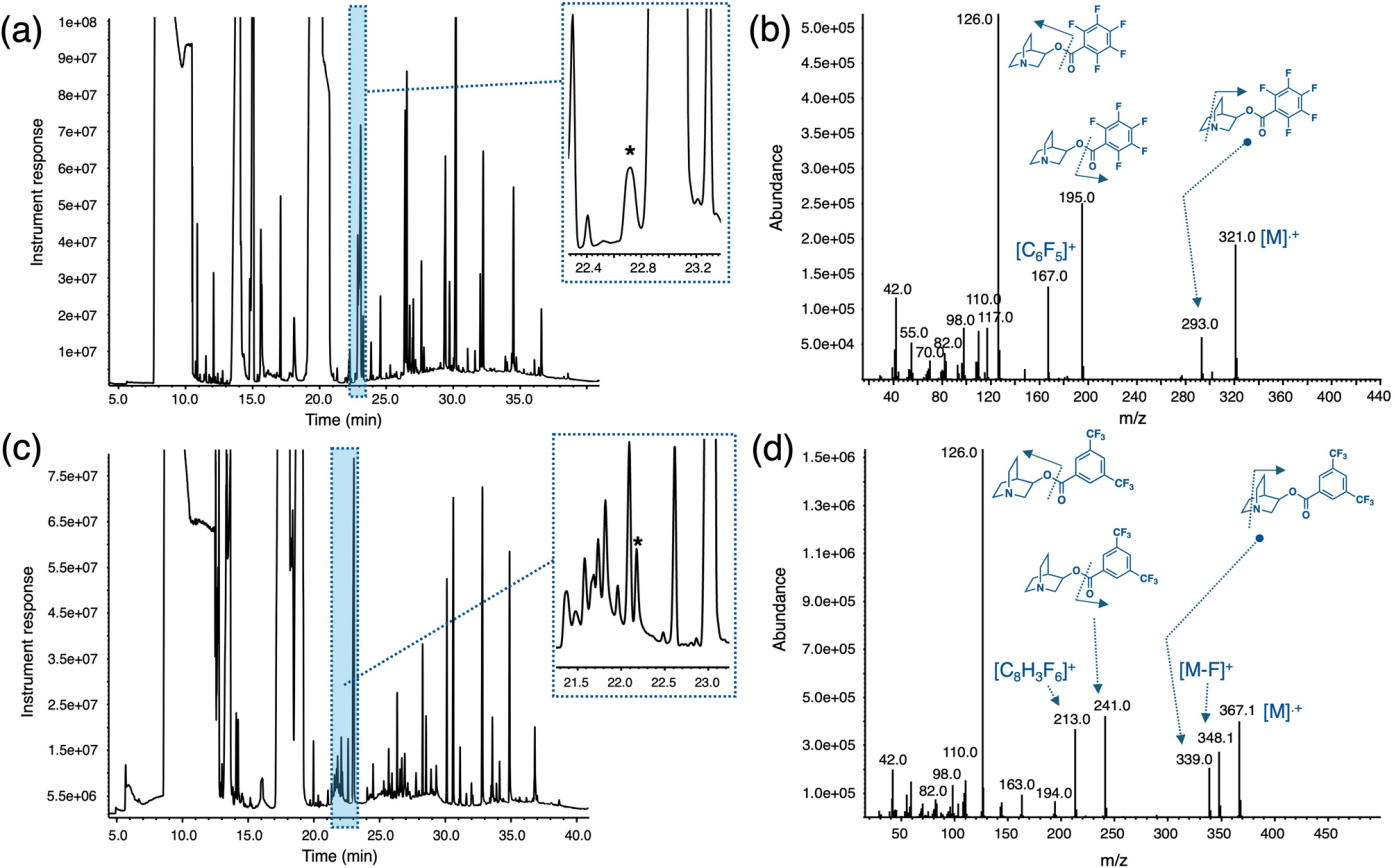
(**a**) Pentafluorobenzoylation of extract from L2 aqueous matrix (3Q spiked at a ~ 50 ppm concentration) to yield derivative 3Q-PFBz featuring a retention time of 22.72 min (denoted with an asterisk in inset); (**b**) mass spectrum of 3Q-PFBz from the derivatized mixture showing proposed fragmentation patterns for specific masses; (**c**) 3,5-bis(trifluoromethyl)benzoylation of extract from L1 aqueous matrix to yield derivative 3Q-BTFMBz featuring a retention time of 22.20 min (denoted with an asterisk in inset); (**d**) mass spectrum of 3Q-BTFMBz from the derivatized mixture showing proposed fragmentation patterns for specific masses.

Liquid matrix 2 (L2) constituted a matrix similar to L1 but collected from another location in addition to having 3Q spiked at a much higher concentration than L1 (~ 50 µg/mL). Preliminary analysis of matrix L2 was found to contain low-medium molecular weight species ranging from C4 to C7 and in some cases as tentatively assigned using the NIST library as well as other organic species consisting of esters and phenolic compounds. As for L1, the first derivatization for L2 involved reacting the mixture with pentafluorobenzoyl chloride to give 3Q-PFBz (RT ~ 22.72 min) that was detected without using the single ion extraction mode (Fig. 6a). It can be observed from Fig. 6a, that 3Q-PFBz can still be observed among some of the other interferences present in the L1 matrix and that were extracted along with 3Q, despite the low concentration of the amino alcohol in it. Figure 6b shows the mass spectrum of the 3Q-PFBz that can be obtained successfully by performing a background subtraction in the mass spectrum. The second derivatization involved reacting the mixture with 3,5-*bis*(trifluoromethyl)benzoyl chloride to give 3Q-BTFMBz (RT ~ 22.20 min) (Fig. 6c). Again, 3Q-BTFMBz can easily be detected without the need of the single ion extraction mode and its mass spectrum can be readily identified by performing background subtraction methods away from the interferences that are at similar or higher concentrations (Fig. 6d). In similar fashion to L1 there is the slight deviation in retention time of both derivatives when produced in the L2 matrix. It appears that for L2 this effect is stronger, even though both derivatives are produced from a high concentration 3Q spiked sample.

## Conclusions

In this work, acylation has been proven to be a viable and effective derivatization strategy for 3Q, a marker for the chemical warfare agent BZ, for its detection and analysis by EI-GC-MS. After carrying rounds of optimization on parameters governing the benzoylation reaction of 3Q, the most optimal conditions were used in the pentafluorobenzoyl- and 3,5-bis(trifluoromethyl)benzoylation of 3Q in OPCW PT matrices. Thus, 3Q, present in a ~ 12 µg/g concentration, was effectively converted into its pentafluorobenzoyl- and 3,5-bis(trifluoromethyl)benzoyl derivatives which were effectively detected in a soil matrix after its extraction with DCM. Furthermore, both derivatizations were effectively used in modifying 3Q in two other aqueous matrices, L1 and L2, where 3Q was present at ~ 5 and ~ 50 µg/g concentrations, respectively. For the aqueous matrices, the extraction was necessary, more so than the soil matrix, as water was found to effectively hydrolyze both benzoyl chloride-based reagents. Due to the difficulty associated with the analysis of 3Q by GC-MS means specifically when present at low concentrations (< 5 ppm), the extraction/derivatization protocols described herein should find wide applicability in the analysis of this commonly spiked CWA marker in OPCW proficiency tests.

## Supplementary Information

Below is the link to the electronic supplementary material.

Supplementary Material 1

## Data Availability

Data is provided in the supplementary information files accompanying the manuscript.
