# Supplementary material for: Esterification of 3-quinuclidinol, a marker for the incapacitant BZ, for analysis by EI-GC-MS in OPCW test matrices
Source: Sci Rep. 2025 Nov 6;15:38894. doi: 10.1038/s41598-025-22716-6 (PMC12592362; doi:10.1038/s41598-025-22716-6)
Supplement: Supplementary file 1 — Supplementary Material 1 [file 41598_2025_22716_MOESM1_ESM.docx]

**-Supporting Information-**

**“Chemical Strategies Based on Acylation Reactions for the Analysis of 3-Quinuclidinol, a Marker for the Incapacitating Agent BZ, by Electron Ionization Gas Chromatography-Mass Spectrometry in OPCW Proficiency Test Matrices”**

David S. Cho^1-3^, David Baliu-Rodriguez^1,4^ and Carlos A. Valdez^1-4,^*

*^1^Physical and Life Sciences Directorate, ^2^Forensic Science Center, ^3^Global Security Directorate and ^4^Biosciences and Biotechnology Division, Lawrence Livermore National Laboratory, Livermore, CA, 94550, USA.*

**Table of Contents**

**Content Page**

NMR experimental description 2

Figure S1. ^1^H NMR spectrum for 3Q-Ac 3

Figure S2. Expanded ^1^H NMR spectrum for 3Q-Ac. 3

Figure S3. ^13^C NMR spectrum for 3Q-Ac. 4

Figure S4. ^13^C NMR-DEPT-135 spectrum for 3Q-Ac. 4

Figure S5. ^1^H NMR spectrum for 3Q-Bz. 5

Figure S6. ^13^C NMR spectrum for 3Q-Bz. 5

Figure S7. ^13^C NMR-DEPT-135 spectrum for 3Q-Bz. 6

Figure S8. ^1^H NMR spectrum for 3Q-PFBz. 6

Figure S9. ^13^C NMR spectrum for 3Q-PFBz. 7

Figure S10. Expansion in ^13^C NMR spectrum for 3Q-PFBz. 7

Figure S11. ^13^C NMR-DEPT-135 spectrum for 3Q-PFBz. 8

Figure S12. ^19^F NMR spectrum for 3Q-PFBz. 8

Figure S13. Expansion in ^19^F NMR spectrum for 3Q-PFBz. 9

Figure S14. ^1^H NMR spectrum for 3Q-BTFMBz. 9

Figure S15. Expansion of ^1^H NMR spectrum for 3Q-BTFMBz. 10

Figure S16. Expansion of ^1^H NMR spectrum for 3Q-BTFMBz. 10

Figure S17. ^19^F NMR spectrum for 3Q-BTFMBz. 11

EI-GC-MS Mass Spectrum for 3Q-Ac. 12

EI-GC-MS Mass Spectrum for 3Q-Bz. 12

EI-GC-MS Mass Spectrum for 3Q-PFBz. 13

EI-GC-MS Mass Spectrum for 3Q-BTFMBz. 13

Optimization studies EI-GC-MS results. 14

**Nuclear Magnetic Resonance**. ^1^H NMR (600 MHz), ^13^C NMR (150 MHz) and ^19^F NMR (565 MHz) were all recorded in CDCl_3_. Spectra were obtained using a Bruker Avance III 600 MHz instrument equipped with a Bruker QNP 5 mm cryoprobe (Bruker Biospin, Billerica, MA) at 25.0 ± 0.1°C. ^1^H NMR chemical shifts are calibrated with respect to residual CHCl_3_ centered at 7.26 ppm, whereas for ^13^C NMR, the center peak for CDCl_3_, centered at 77.0 ppm, was used for the calibration. ^13^C NMR-DEPT-135 data is presented so that positive peaks depict C-H and CH_3_ carbons atoms while negative peaks depict CH_2_ carbon atoms in the molecule. Quaternary carbons (e.g., C=O for the acyl group) are not observed in the ^13^C NMR-DEPT-135 experiment.


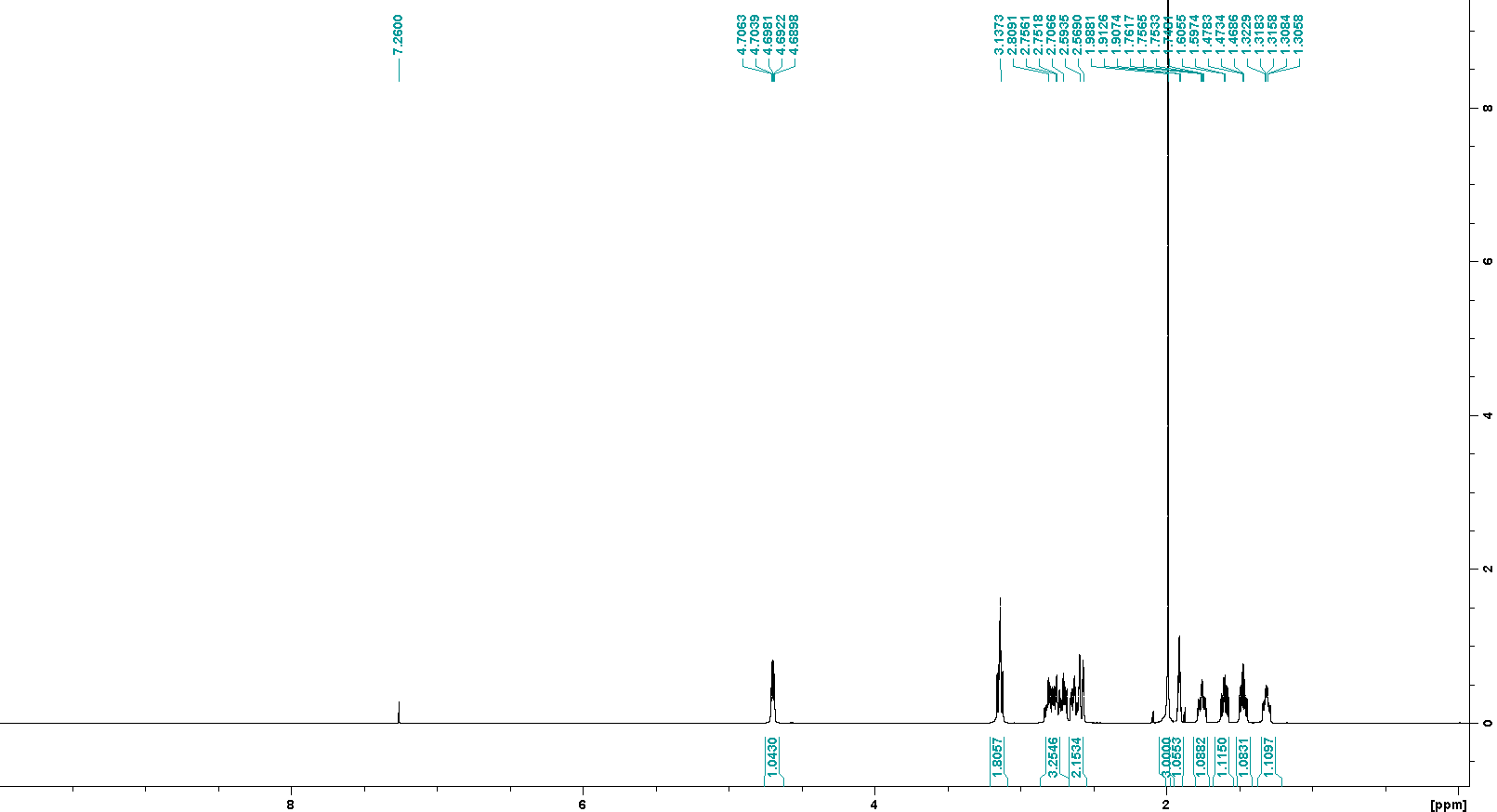


**Figure S1.** ^1^H NMR spectrum for 3Q-Ac (CDCl_3_).


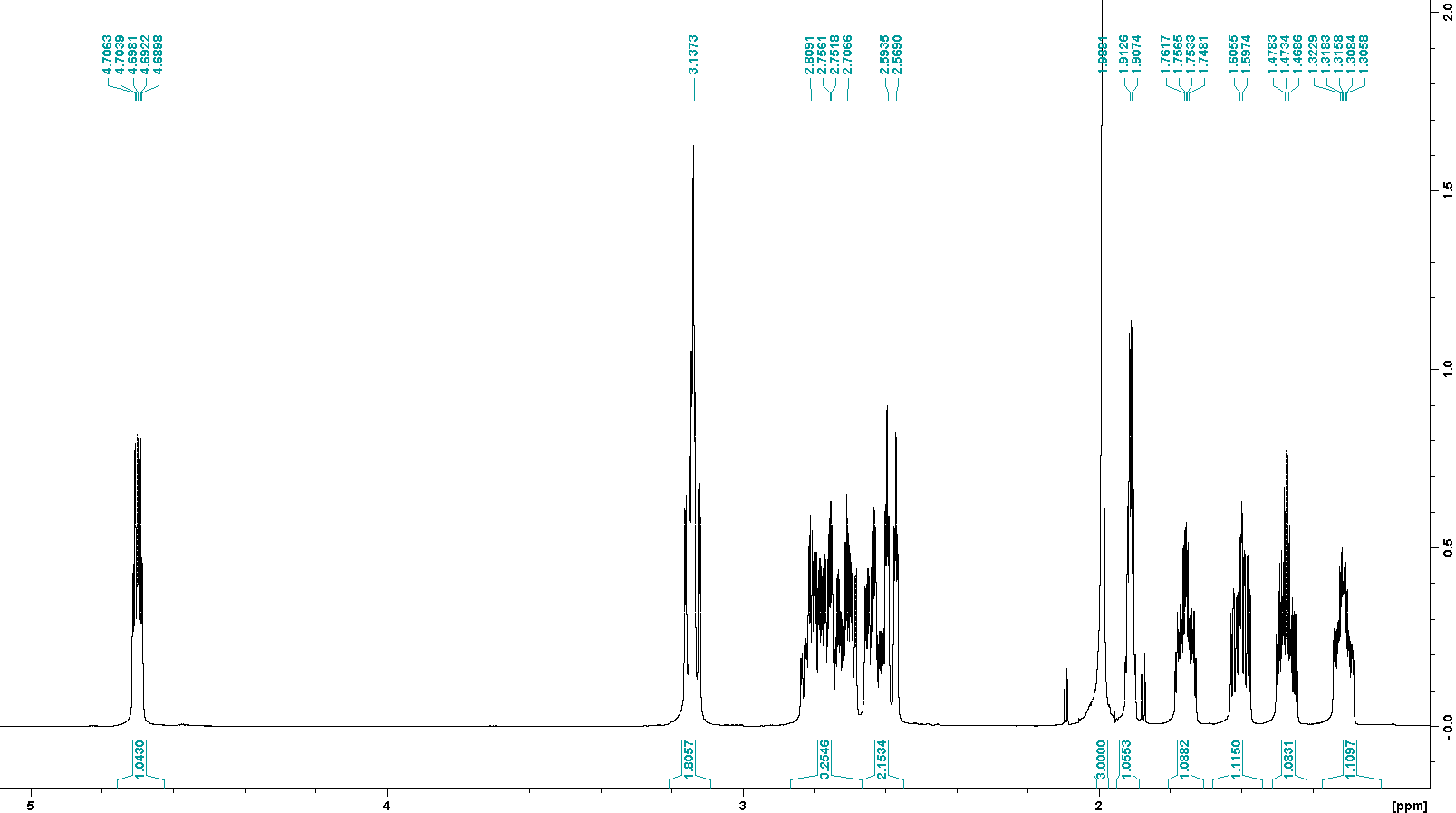


**Figure S2.** Expanded ^1^H NMR spectrum for 3Q-Ac.


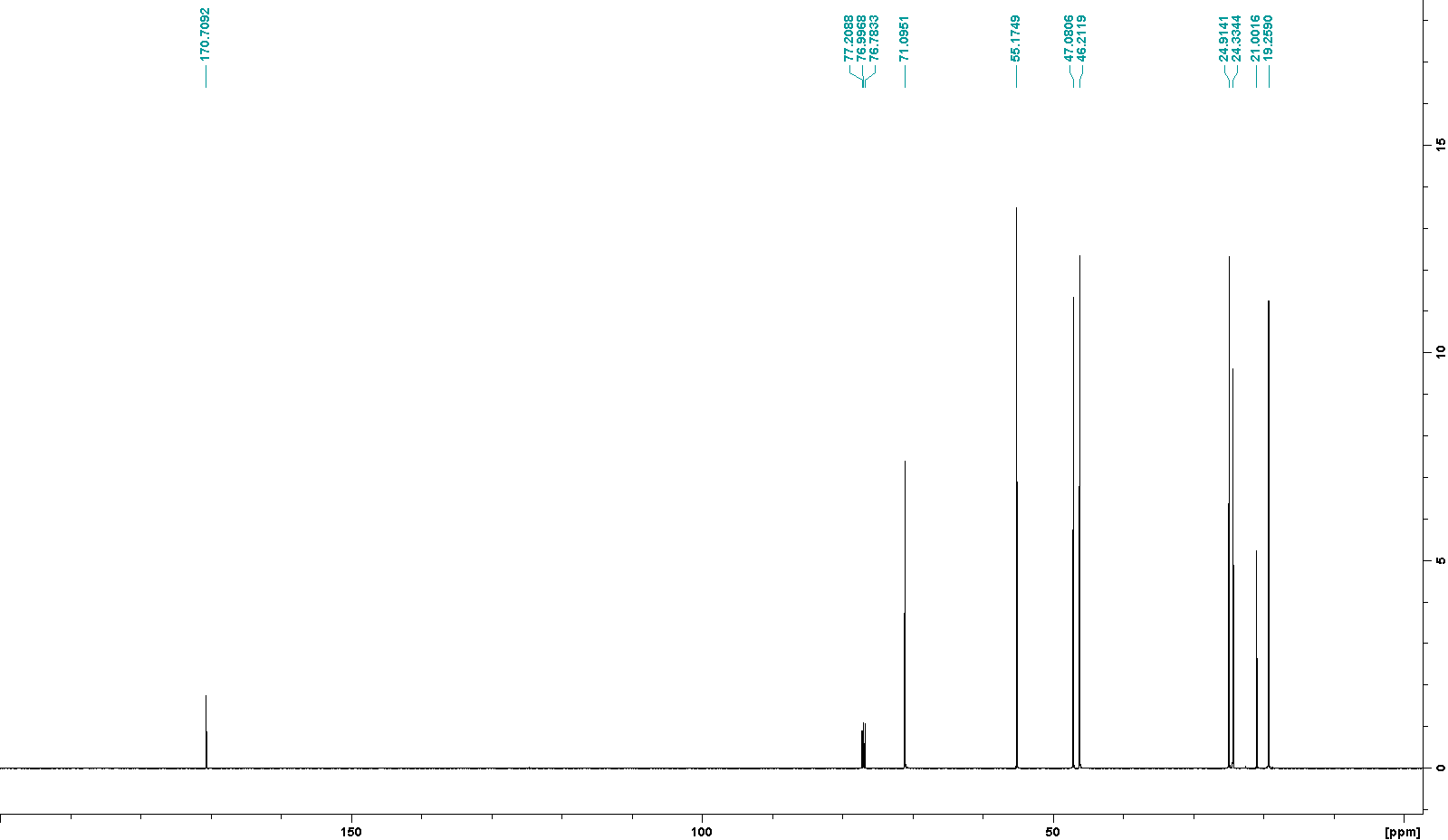


**Figure S3.** ^13^C NMR spectrum for 3Q-Ac.


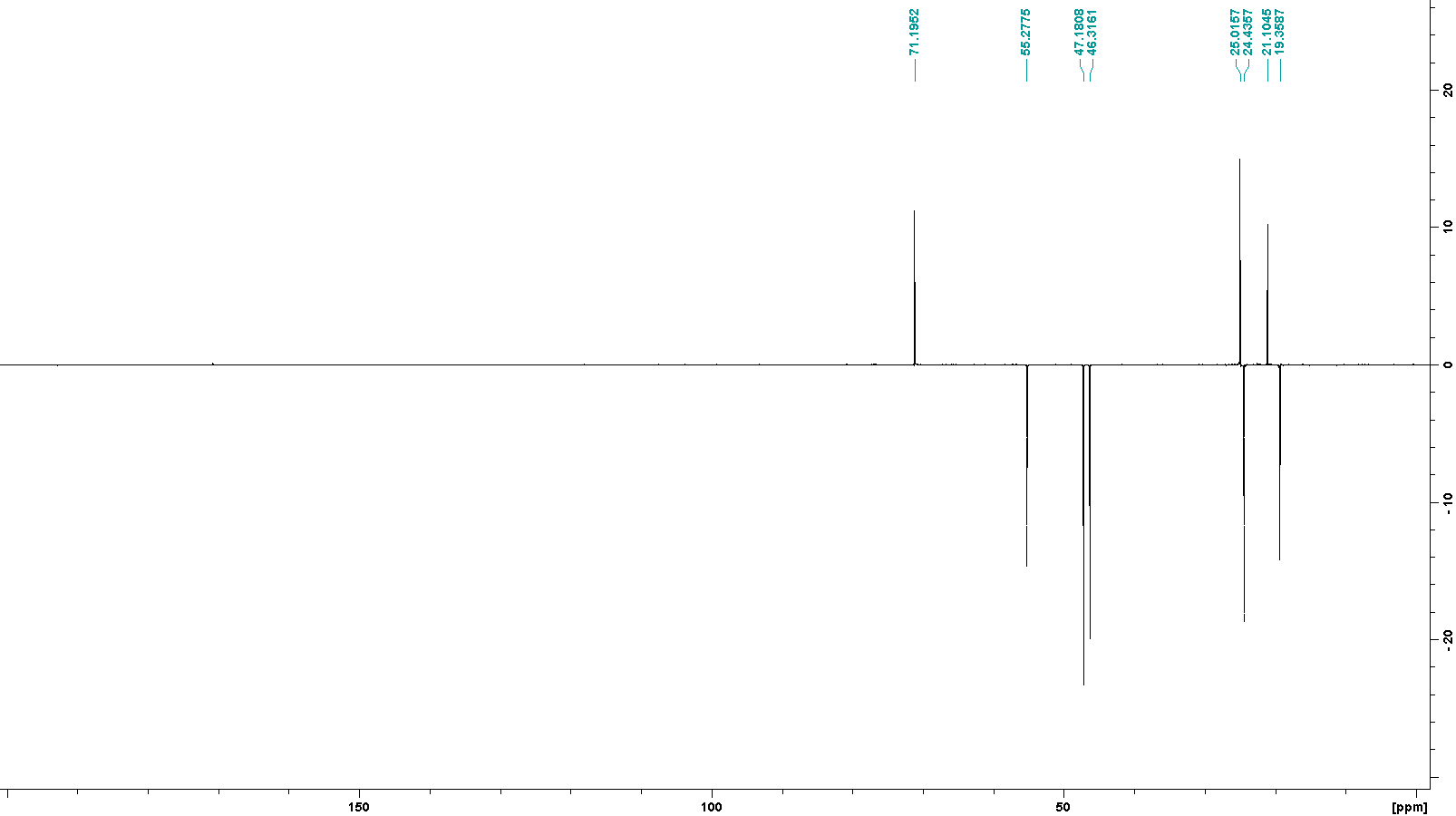


**Figure S4.** ^13^C NMR-DEPT-135 spectrum for 3Q-Ac.


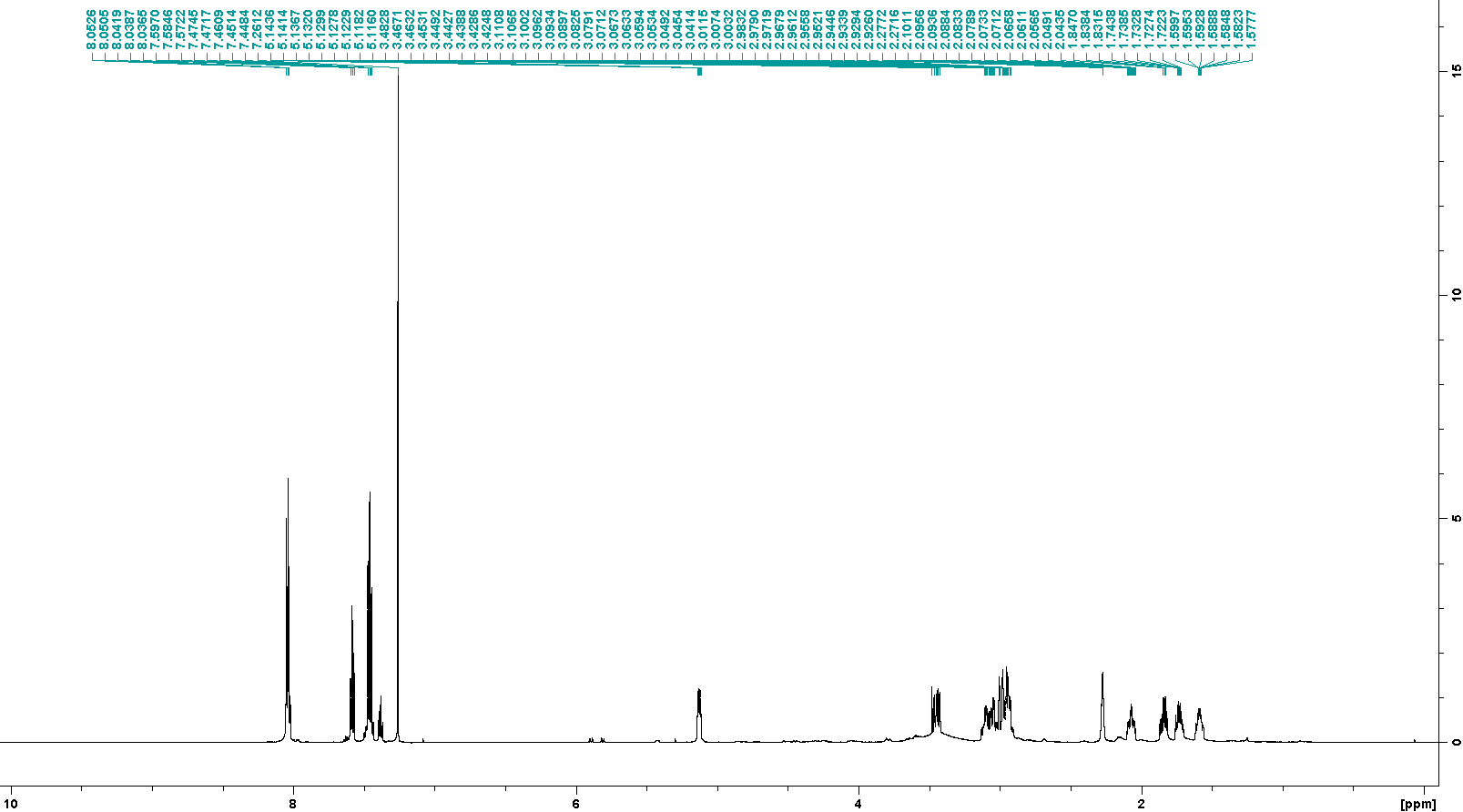


**Figure S5.** ^1^H NMR spectrum for 3Q-Bz (CDCl_3_).


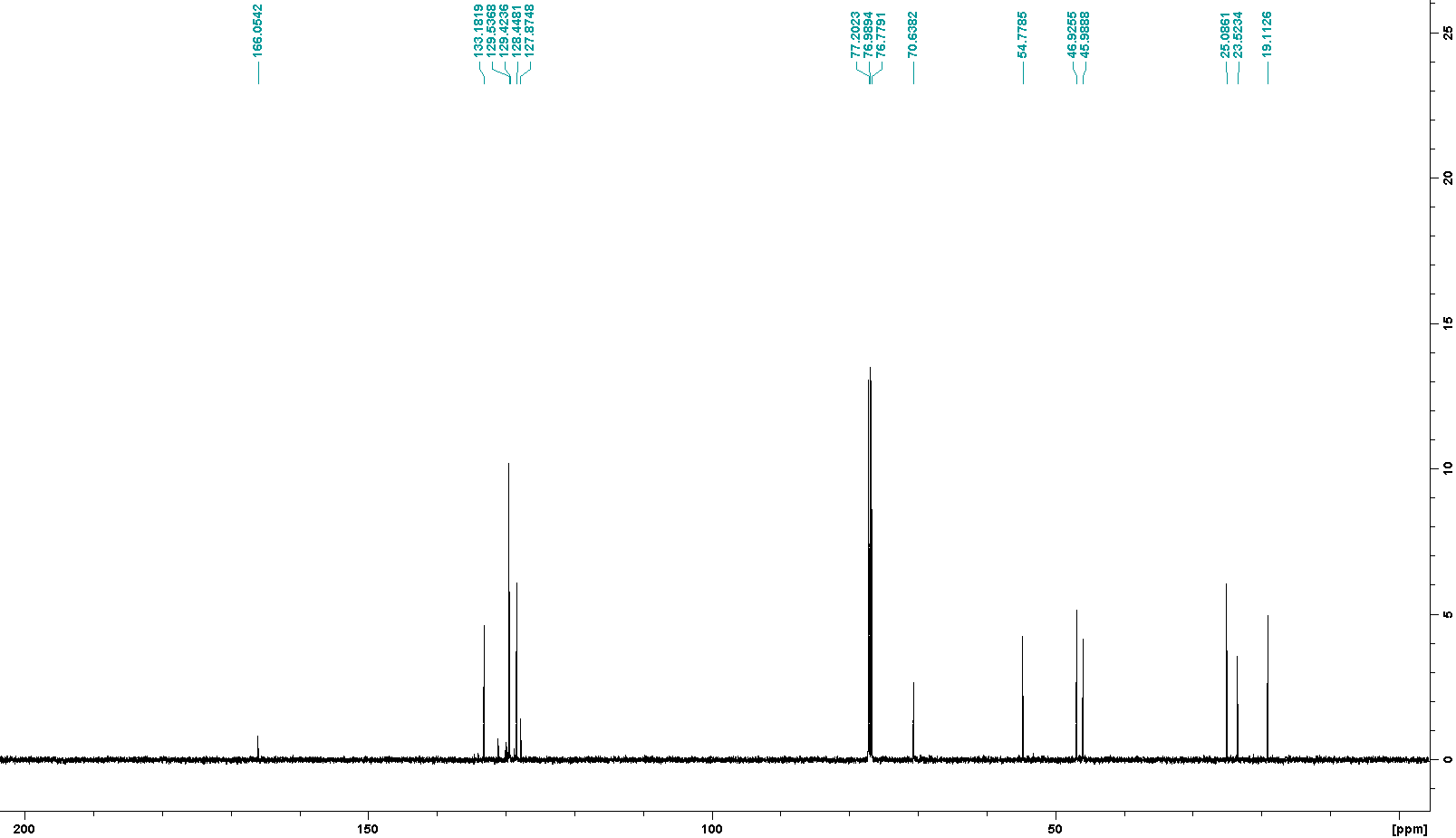


**Figure S6.** ^13^C NMR spectrum for 3Q-Bz.


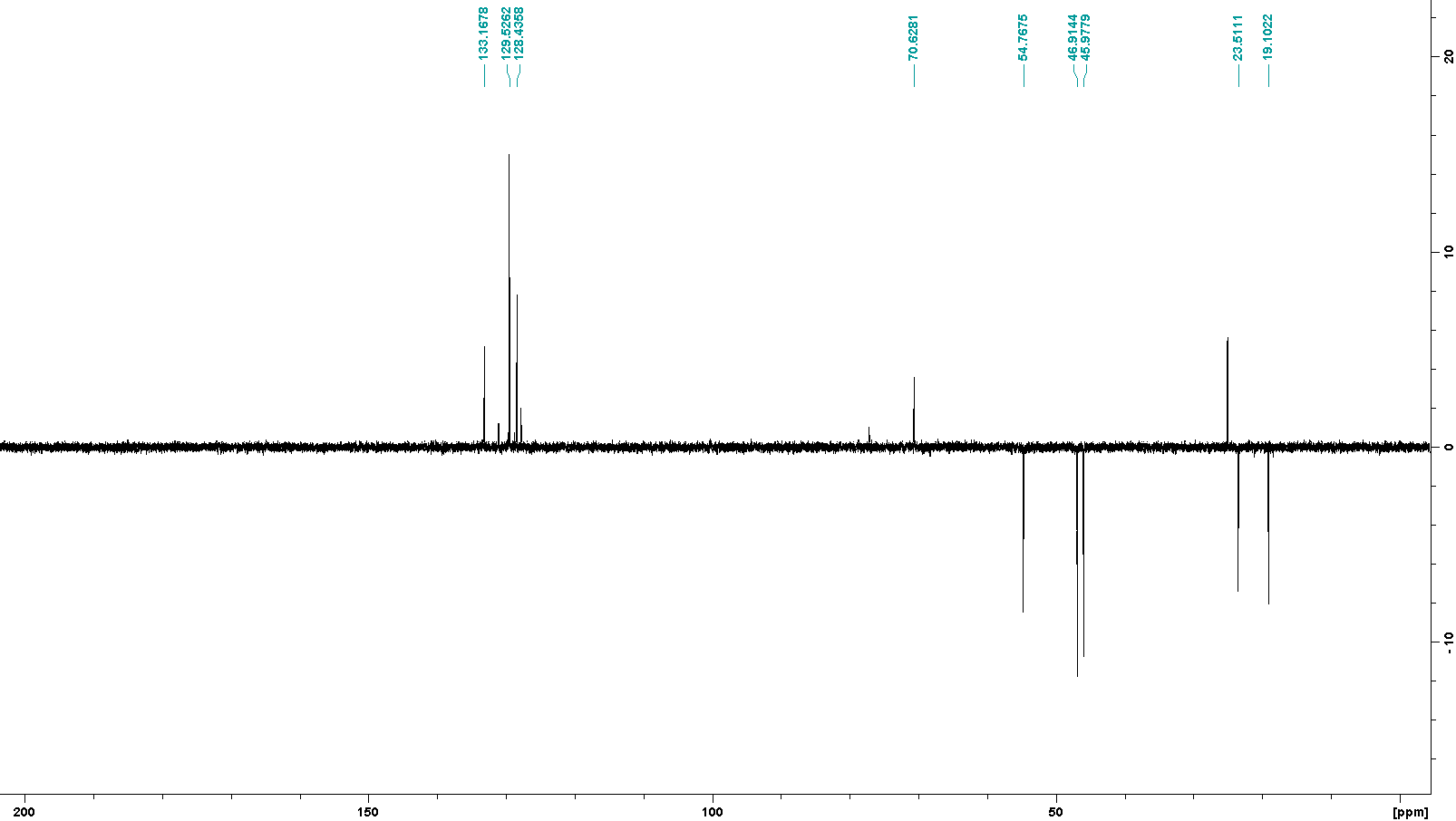


**Figure S7.** ^13^C NMR-DEPT-135 spectrum for 3Q-Bz.


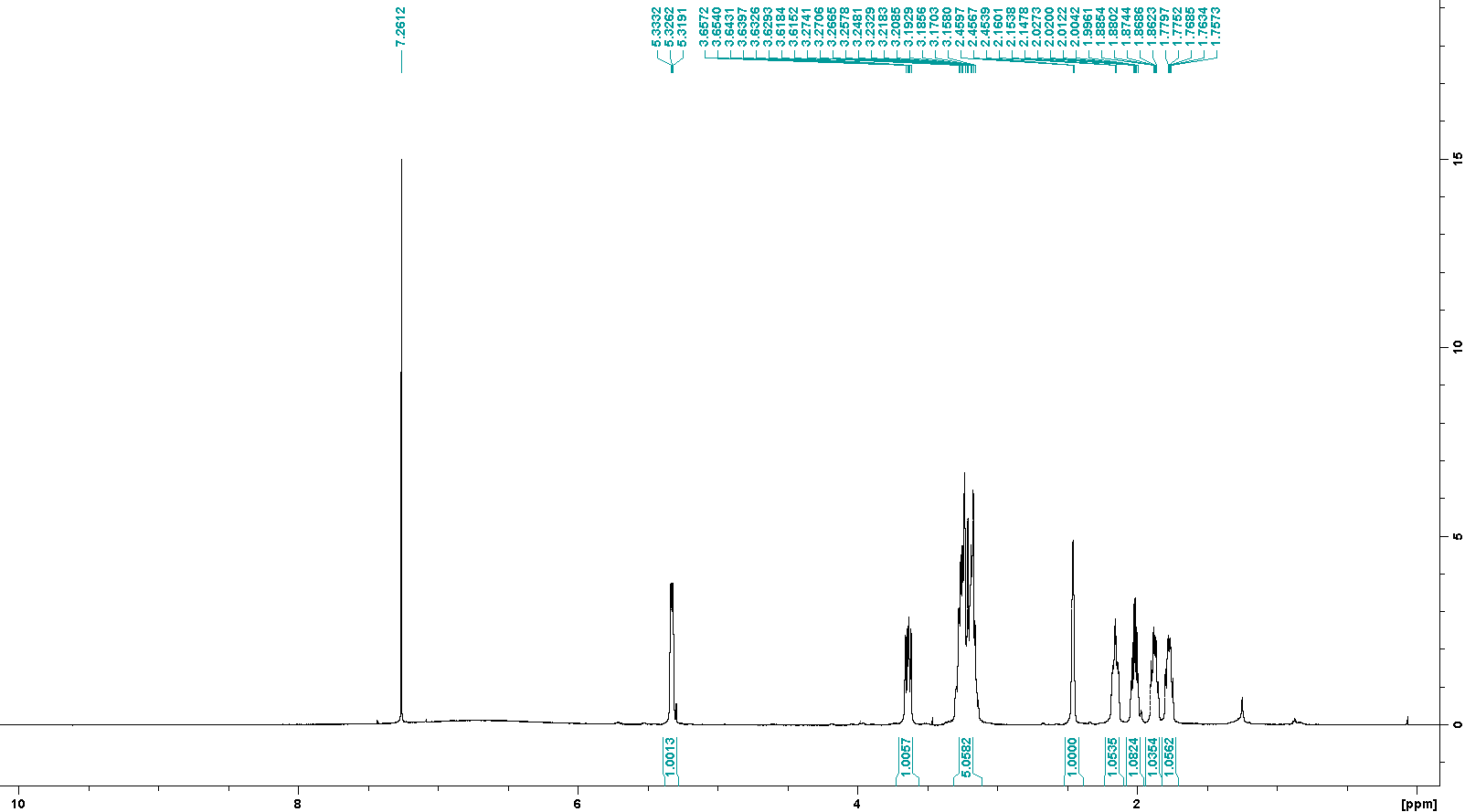


**Figure S8.** ^1^H NMR spectrum for 3Q-PFBz (CDCl_3_).


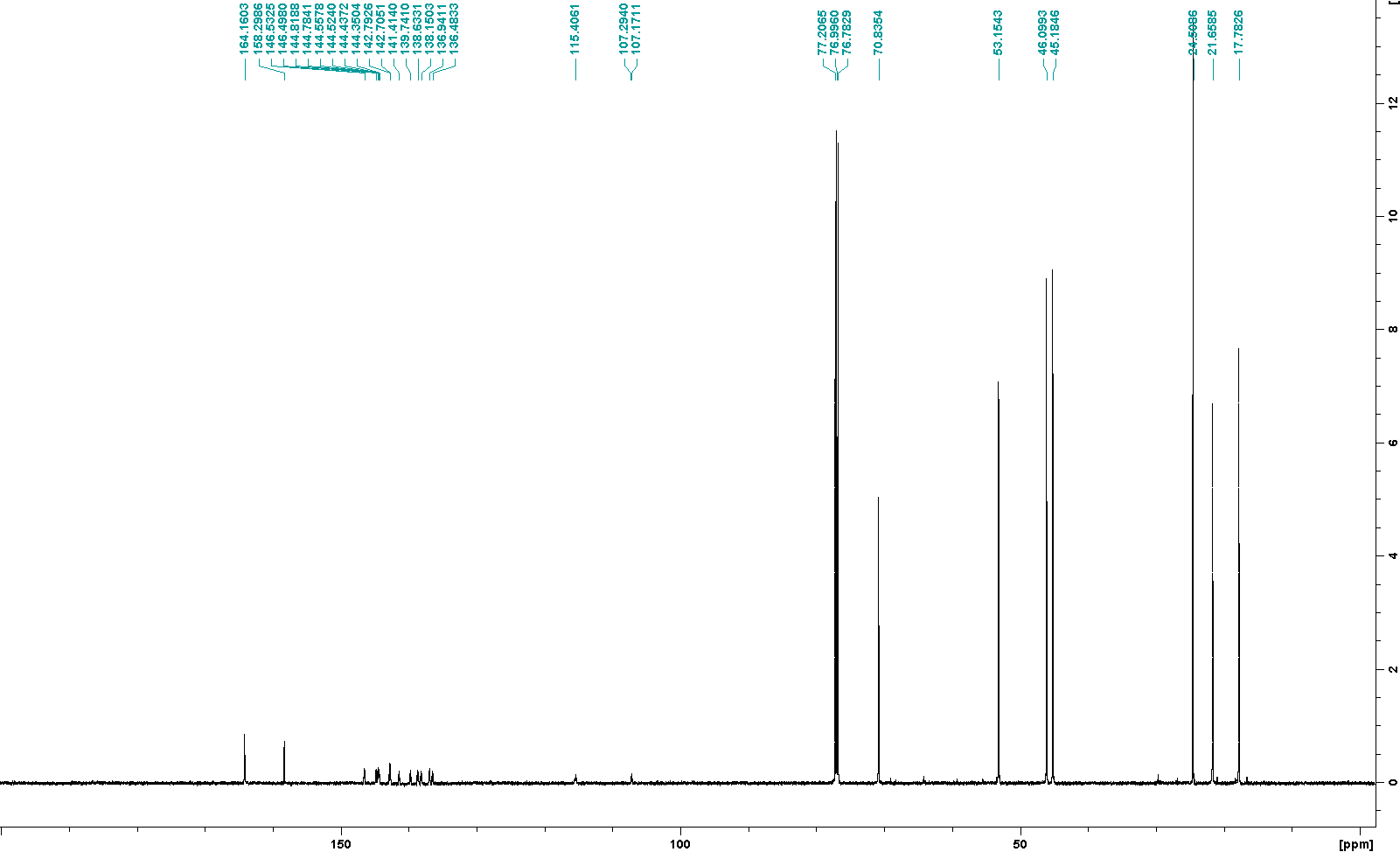


**Figure S9.** ^13^C NMR spectrum for 3Q-PFBz.


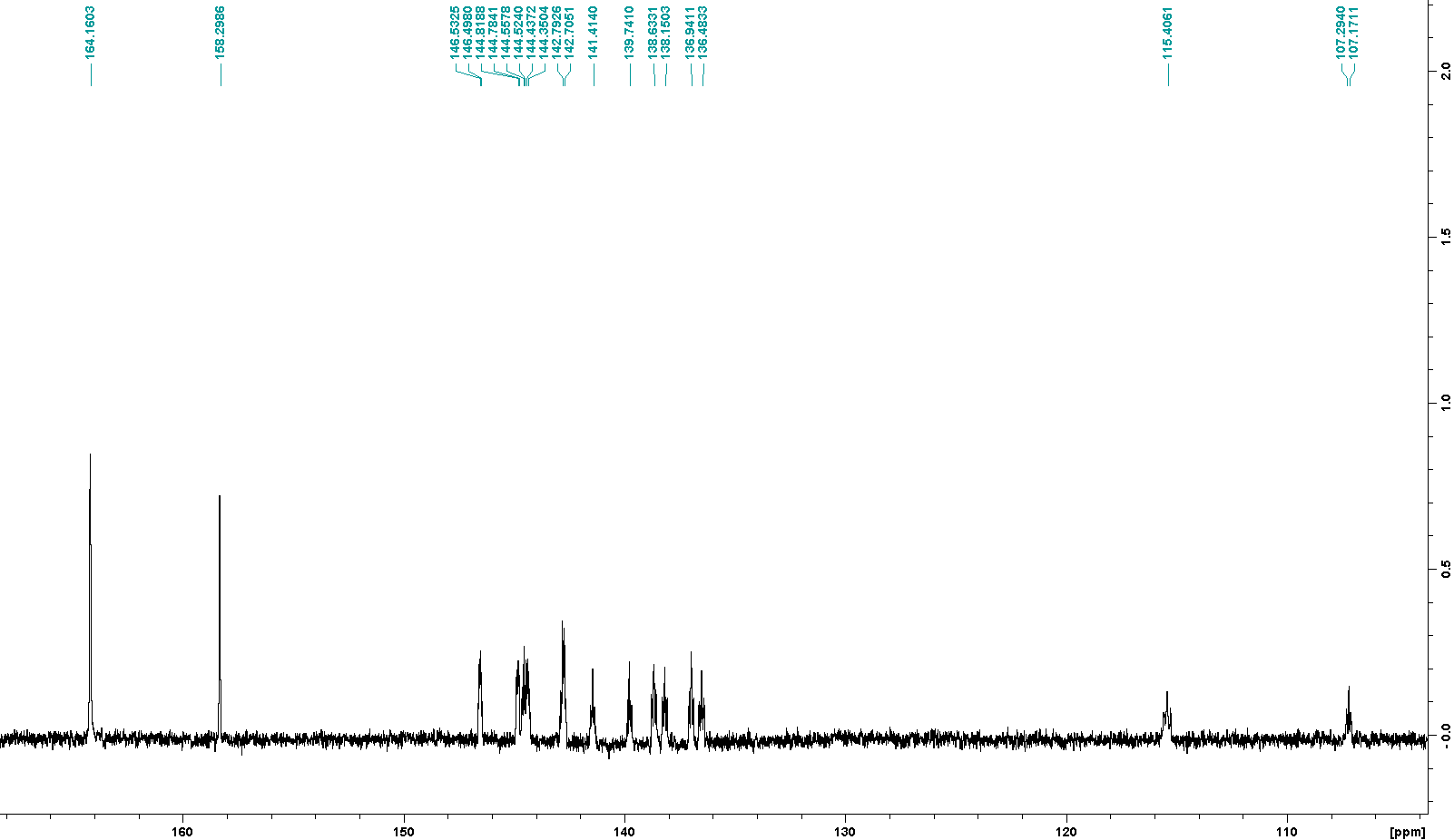


**Figure S10.** Expansion of aromatic region in the ^13^C NMR spectrum for 3Q-PFBz.


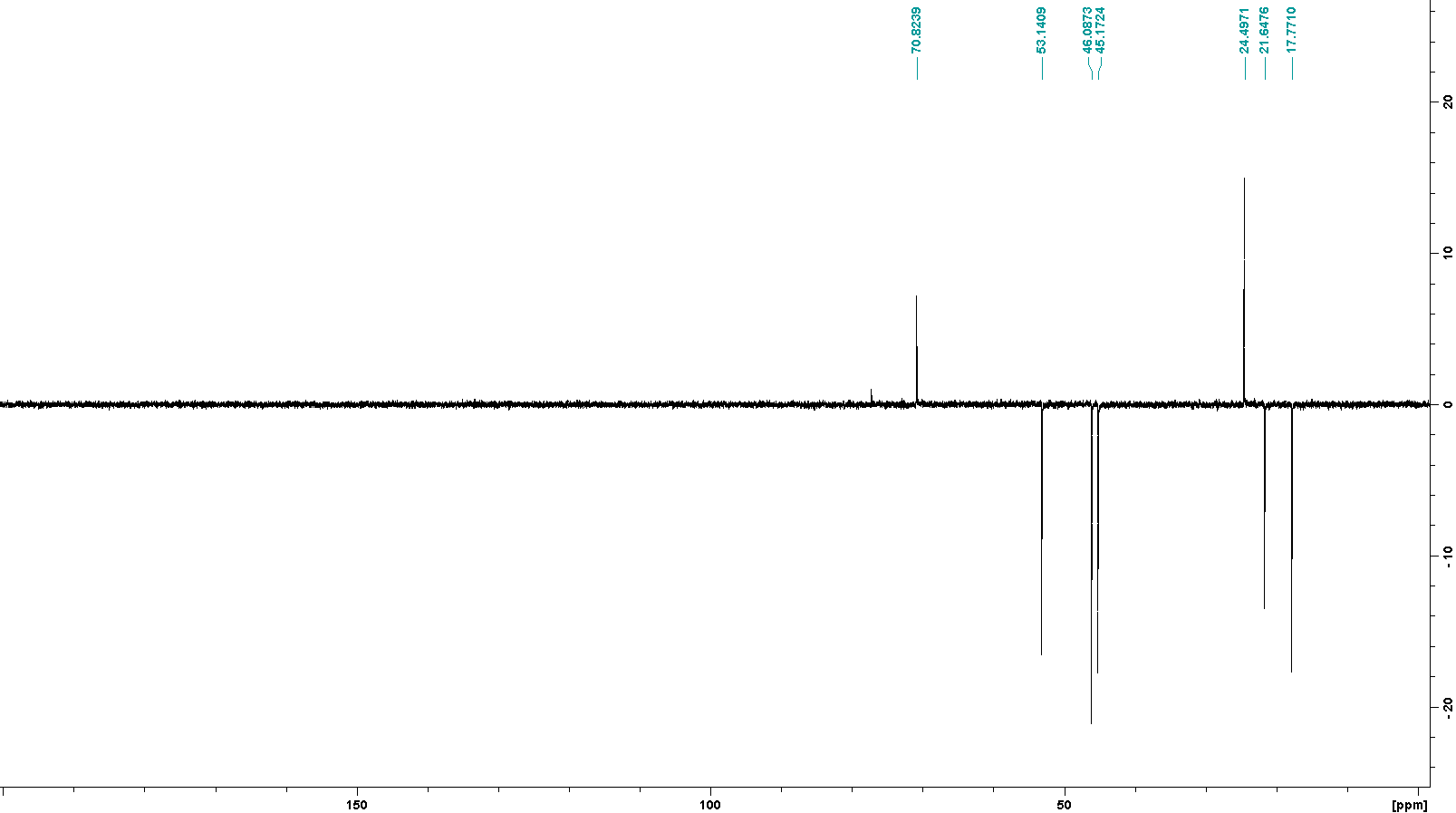


**Figure S11.** ^13^C NMR-DEPT-135 spectrum for 3Q-PFBz.


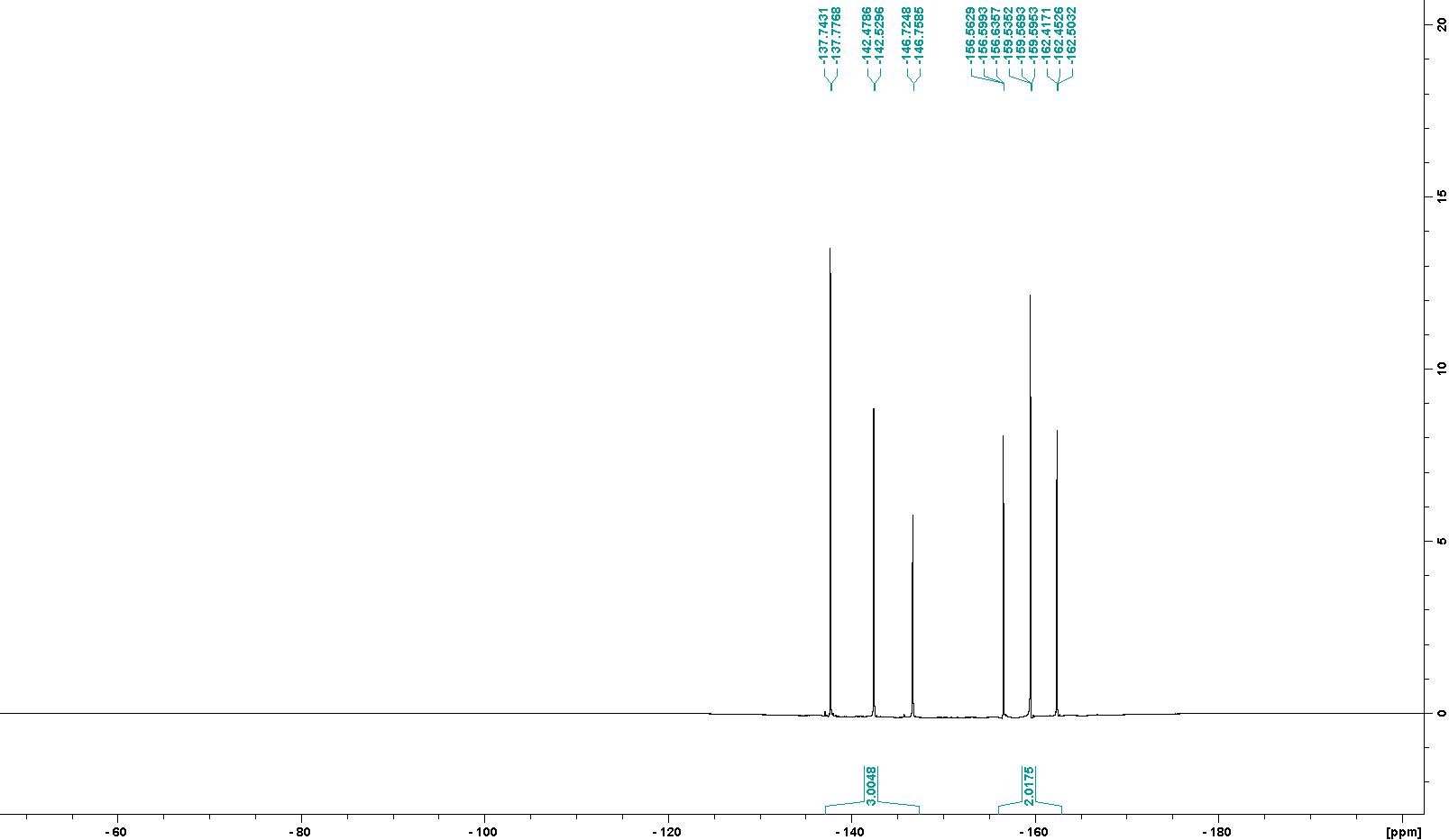


**Figure S12.** ^19^F NMR spectrum for 3Q-PFBz.


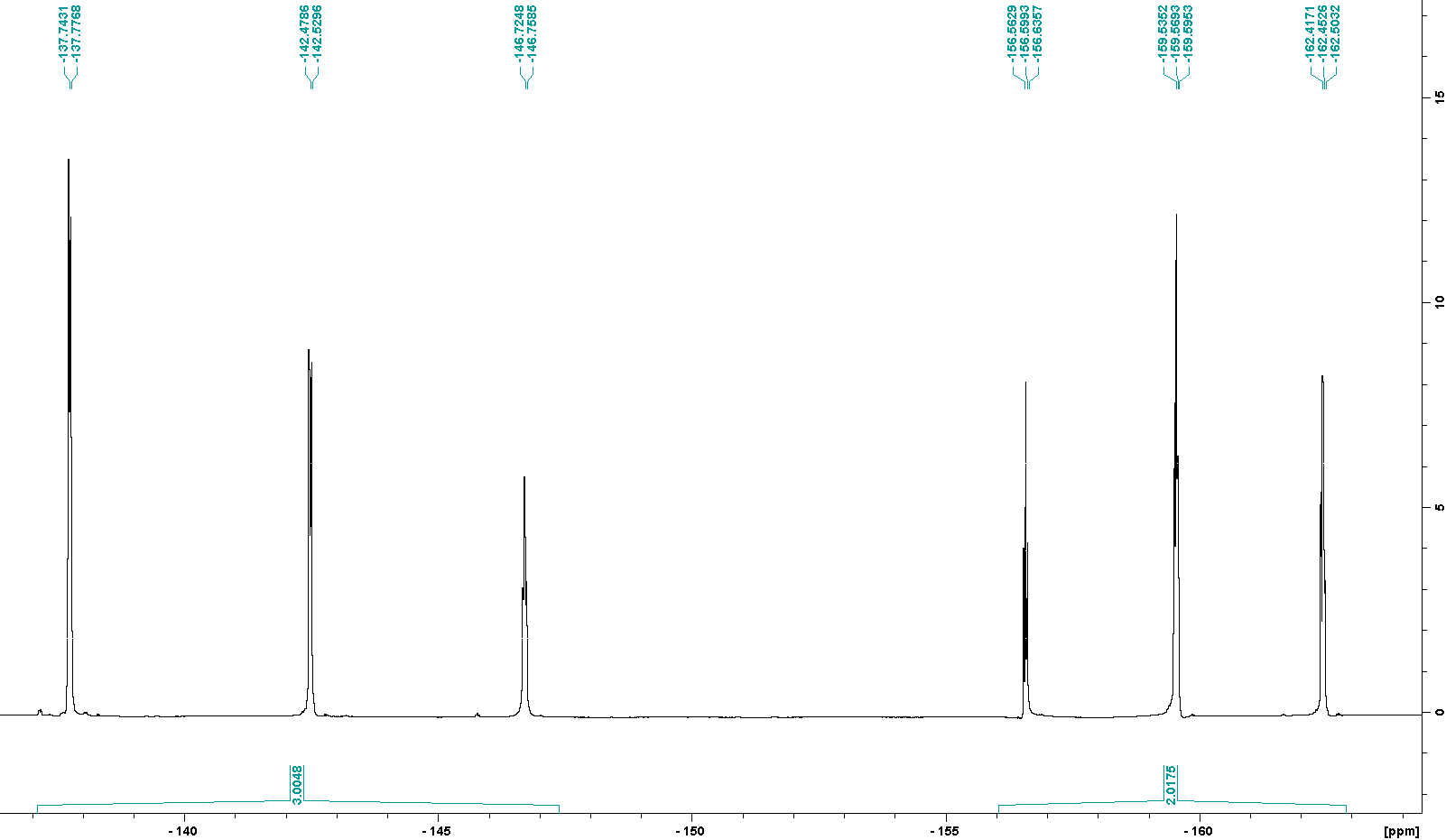


**Figure S13.** Expansion of ^19^F NMR spectrum for 3Q-PFBz.


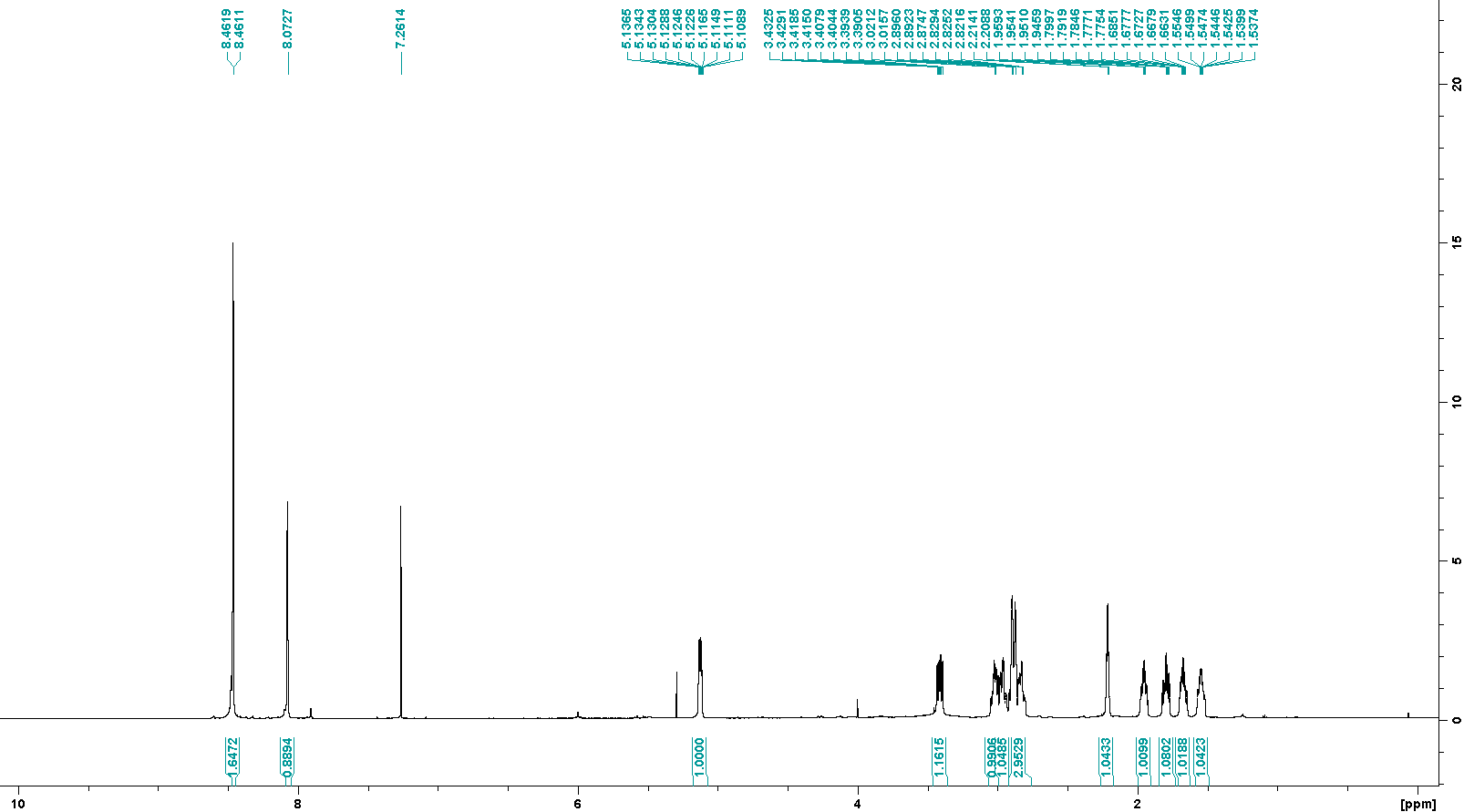


**Figure S14.** ^1^H NMR spectrum for 3Q-BTFMBz (CDCl_3_).


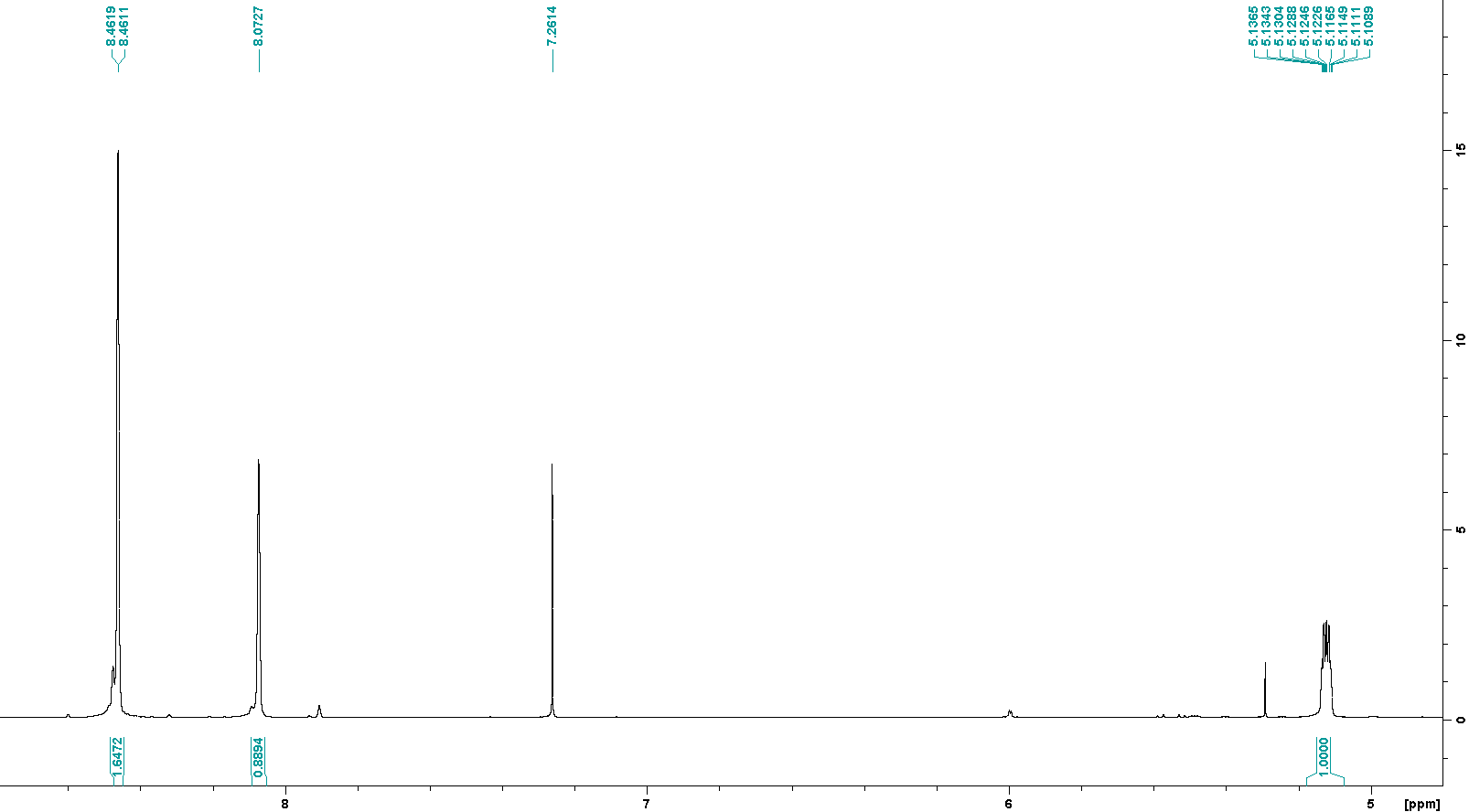


**Figure S15.** Expansion of ^1^H NMR spectrum for 3Q-BTFMBz.


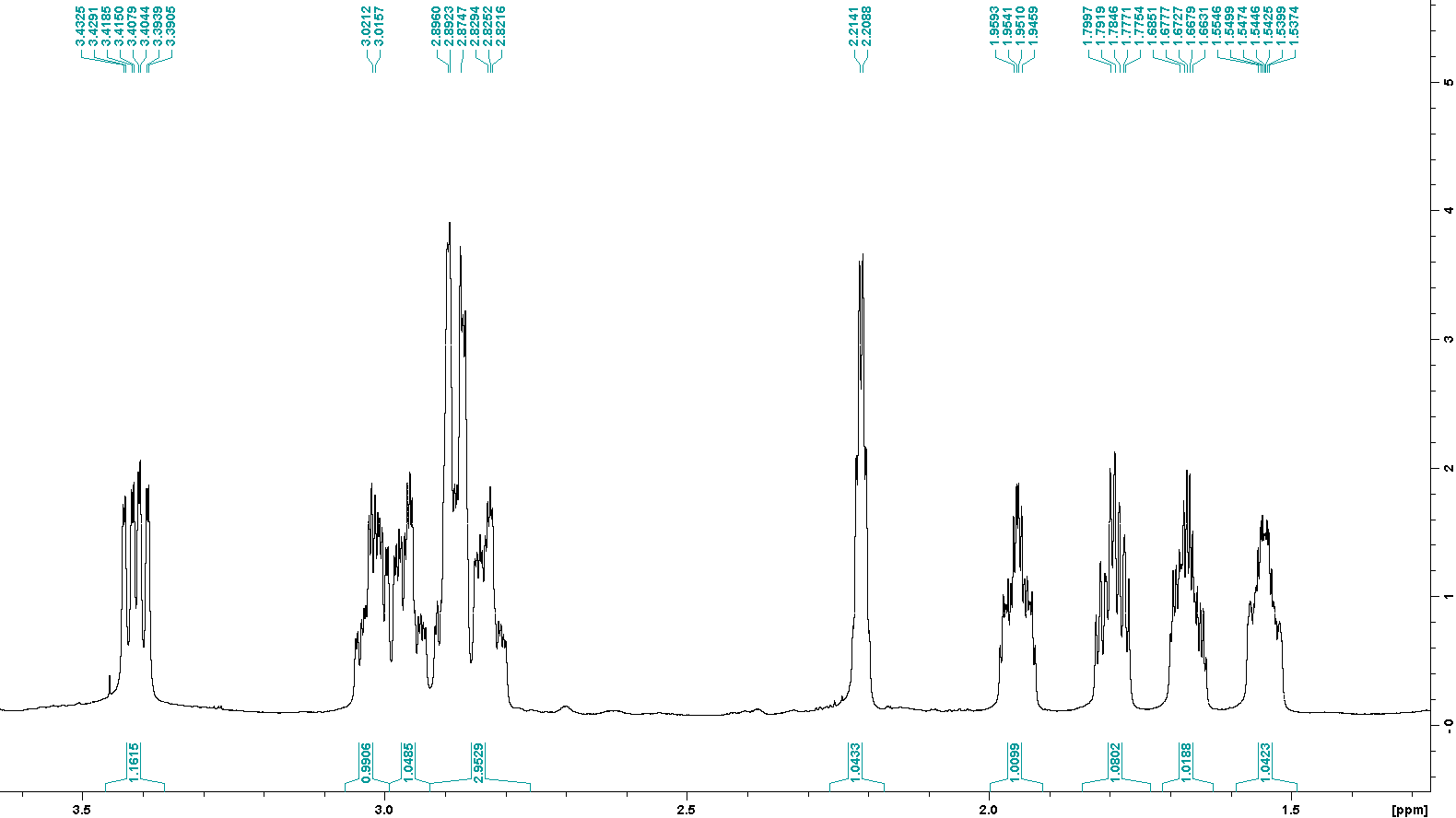


**Figure S16.** Expansion of ^1^H NMR spectrum for 3Q-BTFMBz.


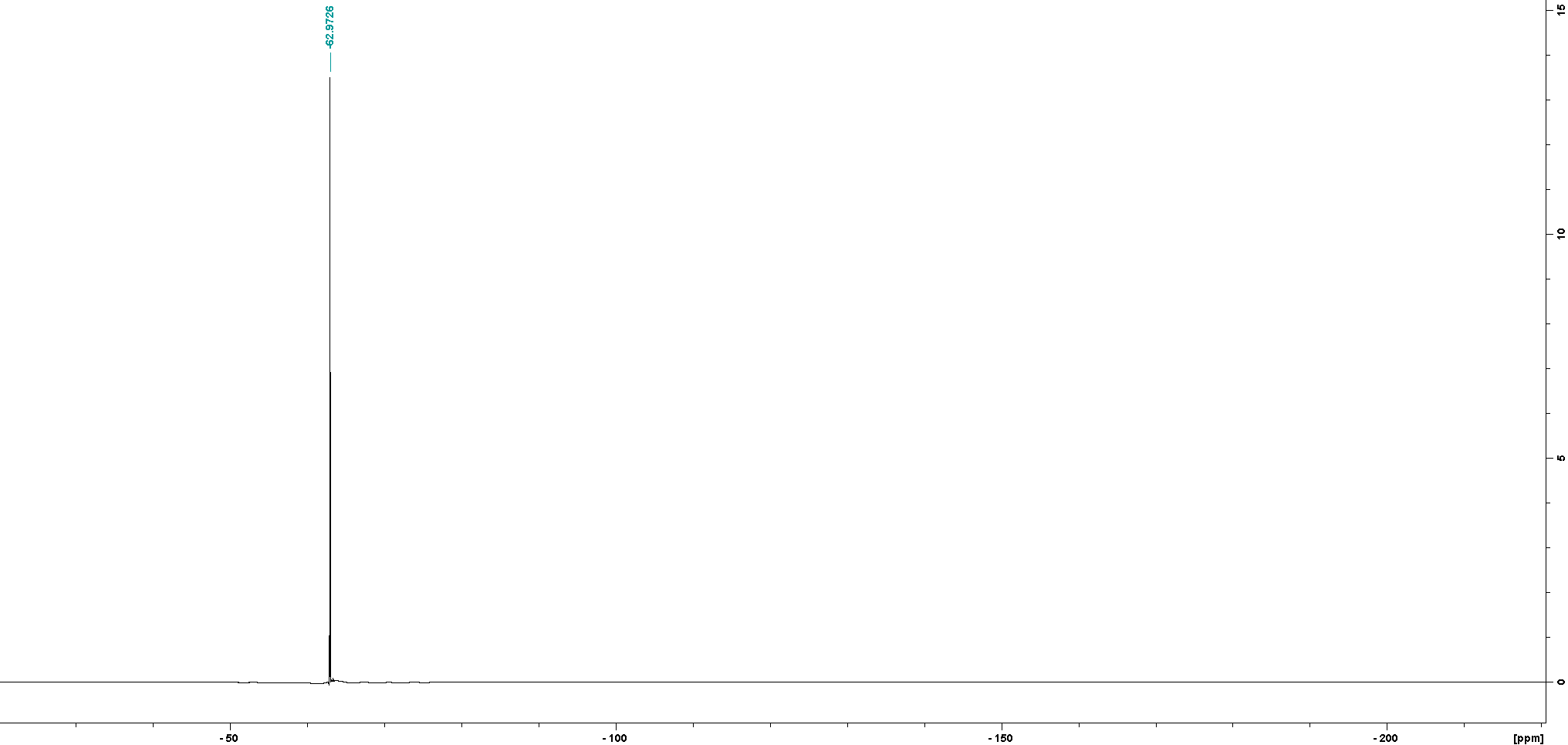


**Figure S17.** ^19^F NMR spectrum for 3Q-BTFMBz.

**Optimization studies performed on the reaction below:**

**Table S1.** Effect of temperature on the benzoylation of 3Q. The number represent the areas under the curve measured for the product 3Q-Bz. A total of six replicates were performed for each temperature under study.

| Temp (^o^C) → | 24 | 40 | 60 |
| --- | --- | --- | --- |
| 1 | 5667 | 8090 | 11367 |
| 2 | 5876 | 8113 | 11989 |
| 3 | 4523 | 8093 | 13421 |
| 4 | 3998 | 7565 | 12177 |
| 5 | 4133 | 6890 | 11993 |
| 6 | 3565 | 6764 | 12710 |

**Table S2.** Effect of time on the benzoylation of 3Q when the reaction occurs at 60 ^o^C. The number represent the areas under the curve measured for the product 3Q-Bz. A total of six replicates were performed for each timepoint under study.

| Time (h) → | 1 | 2 | 3 | 5 | 7 |
| --- | --- | --- | --- | --- | --- |
| 1 | 8889 | 12093 | 13190 | 13212 | 11023 |
| 2 | 9775 | 15411 | 13021 | 13989 | 11434 |
| 3 | 8656 | 11209 | 11546 | 12874 | 12312 |
| 4 | 8003 | 14424 | 14519 | 14778 | 11878 |
| 5 | 9320 | 13457 | 15012 | 14212 | 13612 |
| 6 | 9121 | 14005 | 14998 | 13332 | 12676 |

**Table S3.** Effect of solvent media on the benzoylation of 3Q when the reaction occurs at 60 ^o^C and for 2 hours. The number represent the areas under the curve measured for the product 3Q-Bz. A total of six replicates were performed for each temperature under study.

| Solvent → | CHCl_3_ | DCM | ACN | EtOAc |
| --- | --- | --- | --- | --- |
| 1 | 13244 | 11435 | 9823 | 3112 |
| 2 | 14521 | 12809 | 7786 | 1898 |
| 3 | 11675 | 13132 | 10982 | 1134 |
| 4 | 11645 | 13445 | 7457 | 2454 |
| 5 | 12245 | 12412 | 8867 | 3093 |
| 6 | 12878 | 11778 | 9034 | 3667 |
